# A distal enhancer maintaining Hoxa1 expression orchestrates retinoic acid-induced early ESCs differentiation

**DOI:** 10.1093/nar/gkz482

**Published:** 2019-05-31

**Authors:** Guangsong Su, Dianhao Guo, Jun Chen, Man Liu, Jian Zheng, Wenbin Wang, Xueyuan Zhao, Qingqing Yin, Lei Zhang, Zhongfang Zhao, Jiandang Shi, Wange Lu

**Affiliations:** 1 State Key Laboratory of Medicinal Chemical Biology and College of Life Sciences, Nankai University, 94 Weijin Road, 300071 Tianjin, China; 2 Department of Stem Cell Biology and Regenerative Medicine, Broad Center for Regenerative Medicine and Stem Cell Research, Keck School of Medicine, University of Southern California, Los Angeles, CA 90033, USA

## Abstract

Retinoic acid (RA) induces rapid differentiation of embryonic stem cells (ESCs), partly by activating expression of the transcription factor Hoxa1, which regulates downstream target genes that promote ESCs differentiation. However, mechanisms of RA-induced Hoxa1 expression and ESCs early differentiation remain largely unknown. Here, we identify a distal enhancer interacting with the Hoxa1 locus through a long-range chromatin loop. Enhancer deletion significantly inhibited expression of RA-induced Hoxa1 and endoderm master control genes such as Gata4 and Gata6. Transcriptome analysis revealed that RA-induced early ESCs differentiation was blocked in Hoxa1 enhancer knockout cells, suggesting a requirement for the enhancer. Restoration of Hoxa1 expression partly rescued expression levels of ∼40% of genes whose expression changed following enhancer deletion, and ∼18% of promoters of those rescued genes were directly bound by Hoxa1. Our data show that a distal enhancer maintains Hoxa1 expression through long-range chromatin loop and that Hoxa1 directly regulates downstream target genes expression and then orchestrates RA-induced early differentiation of ESCs. This discovery reveals mechanisms of a novel enhancer regulating RA-induced Hoxa genes expression and early ESCs differentiation.

## INTRODUCTION

Embryonic stem cells (ESCs) have the potential for both self-renewal and differentiation. For normal development, master control genes, which encode transcription factors that directly bind target gene promoters, must be expressed at appropriate stages. Among these are Oct4, Nanog and Sox2, which are essential for the maintenance of pluripotency (1–3), and Gata4 and Gata6, which regulate endoderm development (4–6). Recent studies show that expression of master regulatory genes is controlled by functional elements through long-range chromatin interactions. Functional elements such as enhancers play a key role in maintaining their expression: for instance, Klf4 mediates changes in chromatin structure at the Oct4 locus to maintain ESCs pluripotency, an interaction that enhances reprogramming efficiency (7). Moreover, the Klf4 enhancer maintains naïve pluripotency status of ESCs by regulating Klf4 expression, thus inhibiting ESCs differentiation from a naïve to a primed state (8). Recent studies also show that the Nkx2.5-dependent Gata6 enhancer regulates cardiac development by maintaining Gata6 expression (9). Finally, knockout of multiple Gli3 enhancers promotes abnormal limb development in mouse (10).

Maintenance of RA signaling pathway is essential for ESCs differentiation and organogenesis (11–14). Previous studies have focused more on the activation of master control genes required for RA signaling pathway. However, the role of enhancer in regulating RA signaling pathway remains unexplored. Hoxa1 is a master control gene of RA signaling pathway. Early studies showed that Hoxa1, which has also been called ERA1 (for early retinoic acid 1), is RA-induced and required for embryogenesis and functions in formation of the brain, the inner ear and the cardiovascular system (15–19). Hoxa1 protein interacts with Pbx1/2 and Meis proteins to form a complex that maintains expression of the RA-synthesizing enzyme Raldh2 by directly binding to a Raldh2 functional element. This activity controls RA synthesis and is required for posterior brain development (20,21). Hoxa1 regulates gene expression mainly through directly binding to target promoters and activating specific signaling pathways (15,22–24). Previous studies indicate that an RA response element (RARE) at the Hoxa1 3′-end is necessary for high Hoxa1 expression induced by RA (25,26). Recent studies also report that multiple enhancers located downstream of Hoxa1 are necessary for RA-dependent induction of Hoxa cluster genes (27–29). However, functional enhancers directly regulating Hoxa1 expression over the course of RA-induced ESCs differentiation remain largely unknown. Here, we used Hoxa1 as a model to study the role of enhancer in early differentiation of ESCs induced by RA.

In this study, we employed Capture-C methodology (30,31) to identify functional elements regulating Hoxa1 expression and identified a distal enhancer located at 150 kb downstream of Hoxa1. CRISPR/Cas9-mediated deletion of this enhancer significantly inhibited RA-induced Hoxa1 expression. Transcriptome analysis revealed that RA-induced ESCs differentiation was blocked by enhancer deletion. In addition, ∼40% of genes whose expression changed following enhancer deletion were significantly rescued by Hoxa1 overexpression in enhancer knockout cells, confirming that the enhancer regulates RA-induced ESCs early differentiation by directly regulating Hoxa1. We also show that ∼18% of rescued genes were directly regulated by Hoxa1, among them Crabp1 (Cellular retinoic acid-binding protein 1) and Tcl1 (T-cell lymphoma breakpoint 1). Our study reveals overall that a distal enhancer regulates RA-induced ESCs early differentiation mainly through maintaining Hoxa1 expression.

## MATERIALS AND METHODS

### Cell culture

Mouse ESCs E14 were grown in culture dishes coated with 0.1% gelatin (Sigma) in Glasgow Minimum Essential Medium (GMEM, Gibco) supplemented with 15% fetal bovine serum (FBS, AusGeneX), 100 nM nonessential amino acids (Gibco), 1% sodium pyruvate (Gibco), 200 mM glutamate (Gibco), 1% penicillin-streptomycin (Gibco), 50 μM β-mercaptoethanol (Sigma), 10 ng/ml LIF (ESGRO), 1 μM PD0325901 (a MEK inhibitor, Selleckchem) and 3 μM CHIR99021 (a GSK inhibitor, Selleckchem). The medium was replaced every 2–3 days (32,33). Cells are maintained at 37 °C in a 5% CO_2_ incubator.

### RA-induced ESCs differentiation

For RA treatment, ESCs were induced to differentiate by LIF/2i withdrawal and addition of 2 μM RA (Sigma). Culture medium was replaced everyday.

### RNA extraction, reverse transcription and quantitative Real-Time PCR (qRT-PCR)

Total RNA was extracted from cells using TRIzol Reagent (Life Technologies). cDNA synthesis was performed using a PrimerScript™ RT reagent Kit with gDNA Eraser (TaKaRa), according to the manufacturer's instruction. Polymerase chain reaction (PCR) reactions were performed using Hieff™ qPCR SYBR Green Master Mix (YEASEN) and a BioRad CFX Connect Real-Time system. PCR cycling conditions were: 95 °C for 5  min, 40 cycles of 95 °C for 15  s, 60 °C for 15 s and 72 °C for 30  s. A melting curve of amplified DNA was subsequently acquired. Quantification of target genes was normalized to Gapdh expression. Primer sequences are shown in Supplementary Table 1.

### Generation of Skap2 knockdown cell lines

For Skap2 knockdown, short hairpin RNAs (shRNAs) for GFP (control) and Skap2 were designed using an online tool (www.invivogen.com/sirnawizard/design.php). Oligos were then synthesized (Supplementary Table 2) and constructed with the pSUPER-puro system (RNAi System), following the manufacturer's instruction. ESCs were transfected with shRNAs and plated as single cells in 6-well plates. Puromycin was added 24 h after transfection at a final concentration of 5 μM to select stable lines and medium containing puromycin was changed every 2–3 days (7,34). Skap2 knockdown efficiency was evaluated by qRT-PCR and western blotting.

### Western blotting analysis

Western blotting was carried out using the following primary antibodies: Skap2 (sc-398285), Hoxa1 (sc-293257) and αTubulin (sc-5286) from Santa Cruz Biotechnology together with HRP-linked secondary antibodies (Sungene Biotech, LK2003). HRP activity was detected by Luminol HRP Substrate (Millipore, WBKLS0500). Digital images were taken by the automatic chemiluminescence imaging analysis system (Tanon) (35,36). Image J software was used to quantify relative protein levels.

### CRISPR/Cas9-mediated e-site enhancer deletion and Skap2 poly-A knock-in

The CRISPR/Cas9 system (pXRP_001, Addgene # 49535) was used following published protocols (37,38). Briefly, target-specific guide RNAs (sgRNAs) were designed using an online tool (http://crispr.mit.edu/). sgRNAs with the highest score were selected (Supplementary Table 3).

For e-site enhancer knockout, sgRNAs were cloned into a Cas9-puro vector using the Bsmb1 site. ESCs were transfected with two sgRNA plasmids using Lipofectamine 3000 (Life Technologies), and 24 h later cells were treated with 5 μM puromycin for 24 more hours and then cultured in medium without puromycin for another 5–7 days. Individual colonies were picked and validated by gDNA PCR and subsequent Sanger DNA sequencing. Genotyping PCR primers are listed in Supplementary Table 4.

For Skap2 poly-A knock-in, ESCs were transfected with a target sgRNA plasmid and synthetic oligonucleotides using Lipofectamine 3000 and 24 h later treated with 5 μM puromycin for 48 h and then cultured in medium without puromycin for another 5–7 days. Individual colonies were picked and validated by gDNA PCR. Genotyping PCR primers are listed in Supplementary Table 4, and synthetic oligos are listed in Supplementary Table 5 (29).

### Hoxa1 rescue in Enhancer Knockout (EN-KO) cells

Full-length CDS of Hoxa1 was cloned into the pLCH72 vector (Supplementary Table 6). ESCs (EN-KO cells) were transfected with the Hoxa1-vector using Lipofectamine 3000 (Life Technologies) and then 24 h later were treated with media containing 5 μM puromycin until stably-transduced cells were harvested. qRT-PCR and western blotting were used to identify Hoxa1-overexpression cell line.

### Capture-C analysis

Capture-C libraries were prepared as described with minor modifications (30–31,39). Briefly, RA-induced differentiated ESCs were fixed with 1% (vol/vol) formaldehyde for 10 min at room temperature, quenched with 125 mM glycine in phosphate-buffered saline and then lysed in cold lysis buffer (10 mM Tris–HCl, pH7.5, 10 mM NaCl, 5 mM MgCl2, 0.1 mM EGTA, 0.2% NP–40, 1 × complete protease inhibitor cocktail (Roche)). Chromatin was digested with DpnII (New England Biolabs) at 37°C overnight. Fragments were then diluted and ligated with T4 DNA ligase (Takara) at 16°C overnight. Cross-linking was reversed by overnight incubation at 60°C with proteinase K (Bioline). Then 3C libraries were purified by phenol-chloroform followed by chloroform extraction and ethanol-precipitated at −80°C overnight. Sequencing libraries were prepared from 10 μg of the 3C library by sonication to an average size of 200–300 bp and indexed using NEBnext reagents (New England Biolabs), according to the manufacturer's protocol. Enrichment of 2 μg of an indexed library incubated with 3 μM of a pool of biotinylated oligonucleotides (probe sequences are listed in Supplementary Table 7) was performed using the SeqCap EZ Hybridization reagent kit (# 05634261001, Roche/NimbleGen), following the manufacturer's instructions. Two rounds of capture employing 48–72 h and 24 h hybridizations, respectively, were used. Correct library size was confirmed by agarose gel electrophoresis, and DNA concentrations were determined using a Qubit 2.0 Fluorometer (Thermo Fisher Scientific). All sequencing was performed on Hi-Seq 2500 platforms using paired 150 bp protocols (Illumina).

Capture-C data were analyzed using the method published by James Davies (30). Briefly, the clean paired-end reads were reconstructed into single reads using FLASH (40). After in silico DpnII digestion using the DpnII2E.pl script, the reads were mapped back to the mm10 mouse genome using Bowtie1. At last, chimeric reads containing captured reads and Capture-Reporter reads were analyzed using CCanalyser3.pl. The results can be visualized using Integrated Genome Browser (IGV) (41).

### RNA-seq analysis

Cells were lysed with Trizol reagent (Life Technologies) and RNA was extracted according to the manufacturer's instructions. RNA was then sent to a sequencing company (Novogene) for sequencing. Clean reads were mapped to Ensemble mm10 mouse genome using Hisat2 with default parameters. Gene reads were counted by Htseq. Fold changes were computed as the log2 ratio of normalized reads per gene using DEseq2 R package (42). Genes expression with ∣log2 (fold change)∣⩾ 1 (*P* < 0.05) were considered as significantly altered. Heatmaps were drawn with heatmap2. Two biological replicates were analyzed for each experimental condition.

### Gene ontology (GO) and KEGG pathway analyses

GO and KEGG pathway analyses were performed using the DAVID Functional Annotation Bioinformatics Microarray Analysis tool (http://david.abcc.ncifcrf.gov/) (43,44).

### Published data used in this study

The following published datasets were used in our analysis. GSM2588454 for H3K27ac; GSM2099824 for H3K4me1; GSM2588462 for H3K4me2; GSM2588458 for H3K4me3; GSM2175625 for ATAC-seq; GSM2099823 for Med12; SRX2105296 for Hoxa1 ChIP-seq analyses in mouse ESCs at 24 h after RA induction. GSE96107 for Hi-C; GSM859491 for H3K27ac; GSM2586541 for H3K4me1; GSM881353 for H3K4me2; GSM1258237 for H3K4me3; GSM699165 for CTCF; GSM2645432 for YY1; GSM1439567 for Med1; GSM560345 for Med12; GSM766454 for Smc1a; GSM2111724 for Smc3; GSM1276711 for Pol II; and GSM2267967 for ATAC data in mouse undifferentiation ESCs. Raw reads were aligned using bowtie to build version mm10 of the mouse genome. MACS2 was used to call peaks using a default *P*-value cut-off of 1e-5 (45). Peak annotation was analyzed using ChIPseeker (46). Hi-C data were analyzed using the Hi-C Pro method (47). Domain Caller software was used to predict topologically associating domains (TADs) (48). Interaction maps with 5 kb resolution were produced by the R/Bioconductor package HiTC (49).

### Statistical analyses

Data were analyzed by Student's *t*-test unless otherwise specified. Statistically significant *P*-values are indicated in figures as follows: *P <  0.05, **P <  0.01, ***P <  0.001.

## RESULTS

### Long range chromatin interactions between the Hoxa1 locus and a distal enhancer

**Figure 1.**
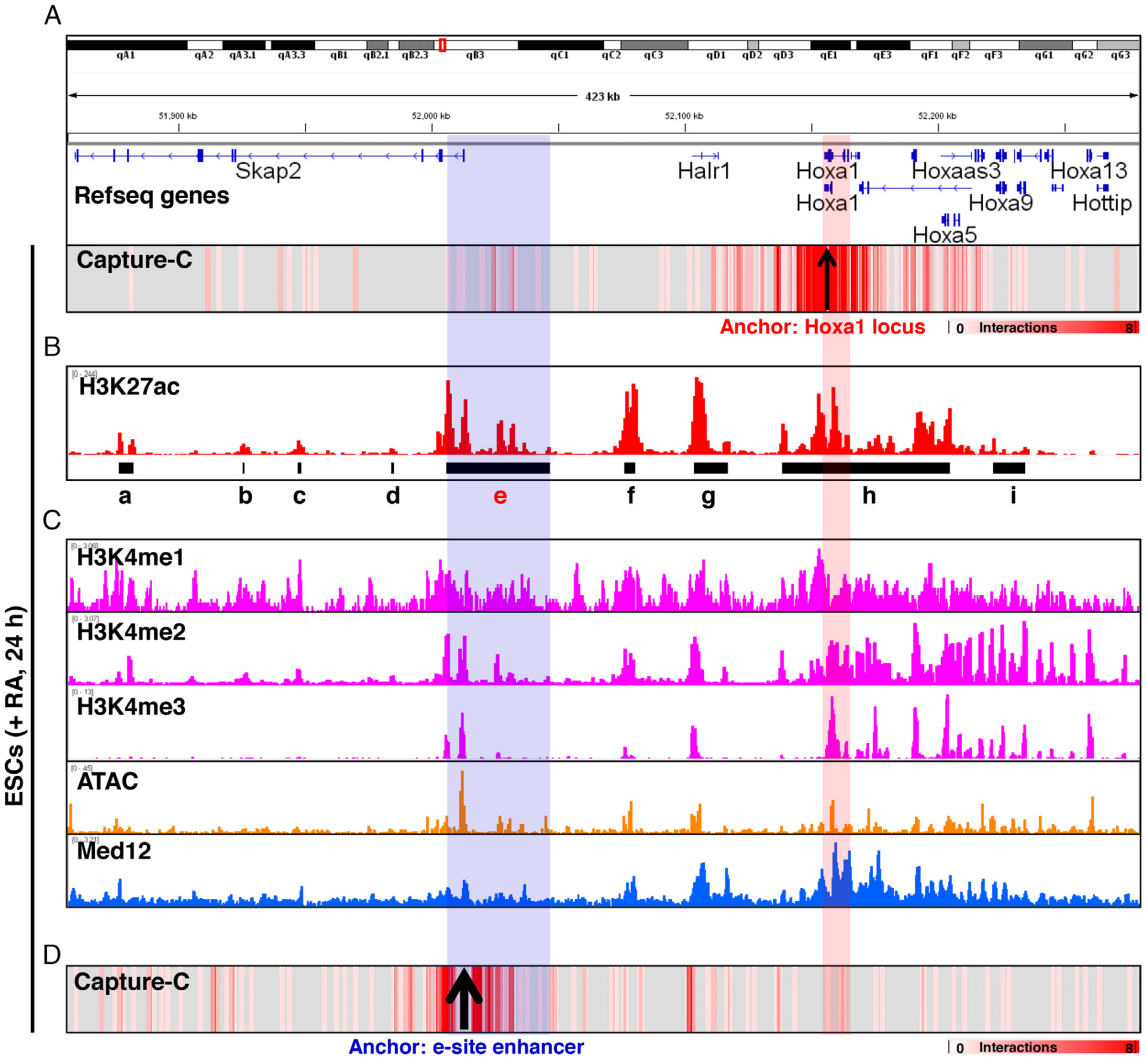
A distal enhancer interacts with Hoxa1 locus through long-range chromatin loop. (**A**) Heatmap illustrating interactions based on Hoxa1 Capture-C in ESCs following RA treatment. (**B**) IGV (Integrative Genomics Viewer) screenshots showing gene tracks of H3K27ac ChIP-seq signal occupancy at Skap2 and Hoxa loci in RA-induced ESCs. Signals of H3K27ac occupancy on the *y*-axis are units of reads per million. Black lines show predicted enhancer regions (Supplementary Figure 1). (**C**) IGV view of selected ChIP-seq tracks at Skap2 and Hoxa cluster loci in RA-induced ESCs. Shown are H3K4me1, H3K4me2, H3K4me3, ATAC-seq and Med12. (**D**) Heatmap illustrating interactions based on e-site enhancer Capture-C in RA-treated ESCs. Blue shadowing indicates e-site enhancer region. Red shadowing shows the Hoxa1 locus.

To identify Hoxa1 enhancers, we used Capture-C methodology (30,31) with the Hoxa1 locus as a bait to capture interacting DNA sequences. To do so, we differentiated ESCs 24 h in the presence of RA and then performed Capture-C and data analysis. Interactions were mainly concentrated in regions adjacent to the Hoxa1 locus (within ∼0.4 Mb) (Figure 1A). Then we analyzed H3K27ac-ChIP data at 24 h after RA induction (27), and 21 983 enhancers were predicted, including 146 super-enhancers (SE) (Supplementary Figure 1A). At the Hoxa1 and Skap2 loci, the e-site enhancer (Figure 1B and Supplementary Figure 1B) among nine predicted enhancers was enriched significantly. Consistent with previous studies (27–29), we also identified significant interactions at the g-site. In addition, H3K4me1/2/3 histone marks were also found to be significantly enriched at the e-site enhancer and Hoxa1 locus (50,51), and an assay for transposase-accessible chromatin with high throughput sequencing (ATAC-seq) revealed that e-site enhancer and Hoxa1 locus with increased chromatin accessibility (52). Med12, a subunit of mediator complex mediating chromatin interaction (53,54), was also significantly enriched (50) (Figure 1C).

We have found significant interactions with e-site enhancer from Hoxa1 locus. To further validate this interaction, we used e-site enhancer as a bait and performed Capture-C technique to capture the DNA sequences interacting with it. Our results show that the e-site enhancer interacted significantly with f- and g-site enhancers and with Hoxa1 gene and the whole Hoxa gene cluster chromatins (Figure 1D). Overall, we conclude that the e-site is a novel Hoxa1 enhancer.

Genes in TADs are preferentially regulated by neighboring enhancers (55–58). When we analyzed published Hi-C data in ESCs (59), we found that the e-site enhancer and Hoxa1 were in the same TAD (Supplementary Figure 2). We also found significant epigenetic markers enrichment at the e-site enhancer, such as H3K27ac and H3K4me1/2/3 (Supplementary Figure 3A). ATAC-seq revealed that the e-site enhancer showed relatively increased chromatin accessibility, and the RNA generation marker Pol II was also enriched at the e-site enhancer (Supplementary Figure 3B). Proteins that mediate chromatin interactions, including Med1, Med12, Smc1a, Smc3, CTCF and YY1, also showed significant binding at the e-site enhancer (Supplementary Figure 3C), suggesting that the e-site is also functional enhancer in undifferentiation ESCs.

### e-site enhancer knockout inhibits RA-induced Hoxa1 expression

**Figure 2.**
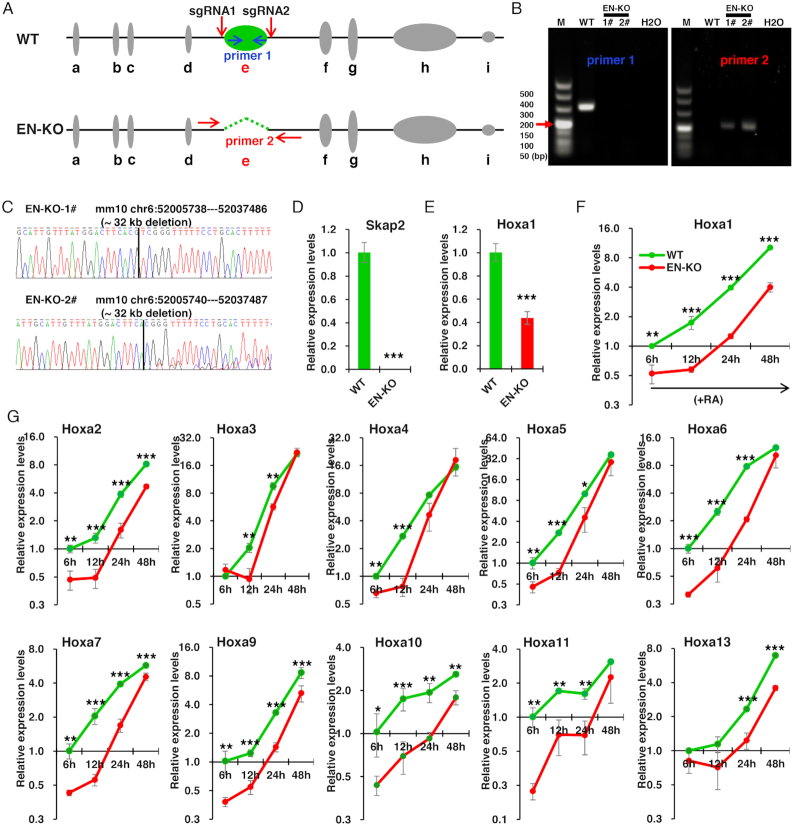
e-site enhancer knockout inhibits RA-induced Hoxa1 expression. (**A**) Schematic showing CRISPR/Cas9-mediated deletion of the e-site enhancer (green) using two sgRNAs. Indicated primers were used to distinguish EN-KO (enhancer knockout) from wild-type (WT) clones. (**B**) Validation of knockout lines by genomic DNA PCR. Shown are images from representative clones. (**C**) DNA sequencing of two EN-KO clones (#1 and #2) using primer 1. (**D** and **E**) Skap2 and Hoxa1 mRNA levels in undifferentiation ESCs, as measured by qRT–PCR and normalized to Gapdh levels. (**F**) Hoxa1 mRNA levels in EN-KO and WT cells over the course of RA-induced ESCs differentiation. mRNA levels were measured by qRT–PCR and normalized to Gapdh levels. (**G**) Hoxa2–a13 mRNA levels were also measured by qRT–PCR and normalized to Gapdh levels in EN-KO and WT cells following RA treatment. Data are represented as mean values ± s.d. Indicated significance is based on Student's *t*-test (*P < 0.05, **P < 0.01, ***P < 0.001). In (D–G), *n* = 3 or 6, including 1 WT, 2 EN-KO cell lines (EN-KO-1# and EN-KO-2#) and three technical replicates per cell line. M: DNA Marker.

To determine whether the e-site enhancer regulates Hoxa1 expression, we performed CRISPR/Cas9 to knock out the e-site enhancer, resulting in identification of two enhancer knockout (EN-KO) lines (Figure 2A–C). Briefly, we designed one sgRNA at each end of the e-site enhancer, and the recombination after cleavage would lead to deletion of the e-site enhancer (Figure 2A). Through gDNA-PCR with specific primers and Sanger sequencing, we confirmed two homozygous cell lines with ∼32 kb of the genome knocked out (Figure 2B and C). Considering the potential miss effect of sgRNAs, we select the predicted miss sites of Top5 for each sgRNA to verify. By designing primers and comparing gDNA-PCR with WT in three cell lines, we found that no genetic changes were found in enhancer knockout cell lines, indicating that there was no miss-target effect of the sgRNA in the two clones we used (Supplementary Figure 4).

In undifferentiation ESCs, Skap2 expression was not detected in EN-KO cells (Figure 2D), as the e-site enhancer partially overlaps with the Skap2 transcription initiation site (TSS). Thus, knocking out the enhancer also knocks out Skap2. We also observed significantly downregulated expression of Hoxa1 in EN-KO cells untreated with RA (Figure 2E), indicating that the enhancer is required for Hoxa1 expression even in an undifferentiated state.

Next, we treated ESCs with RA in differentiation medium to test a requirement for the enhancer for Hoxa1 expression over the course of differentiation. Following RA treatment, we assayed Hoxa1 expression at four time points (namely at 6, 12, 24 and 48 h post-RA treatment). Hoxa1 expression was significantly inhibited in EN-KO relative to WT cells during differentiation (Figure 2F), indicating that the enhancer is required for Hoxa1 expression in this context. In addition, given that Capture-C results of e-site enhancer revealed multiple site interactions with Hoxa cluster chromatins, we speculated that enhancer knockout may also alter expression of Hoxa genes other than Hoxa1. Accordingly, we also observed significant inhibition of Hoxa2–a13 expression in EN-KO cells as compared to WT cells during differentiation (Figure 2G). Moreover, at longer differentiation times, the inhibitory effect gradually decreased (such as Hoxa3, Hoxa4, Hoxa5, Hoxa6 and Hoxa11), indicating that temporal factors regulate enhancer regulation of Hoxa2–a13 expression. Overall, our results revealed that the e-site is a novel Hoxa1 enhancer.

### The expression and transcription of Skap2 are not required for Hoxa1 expression

**Figure 3.**
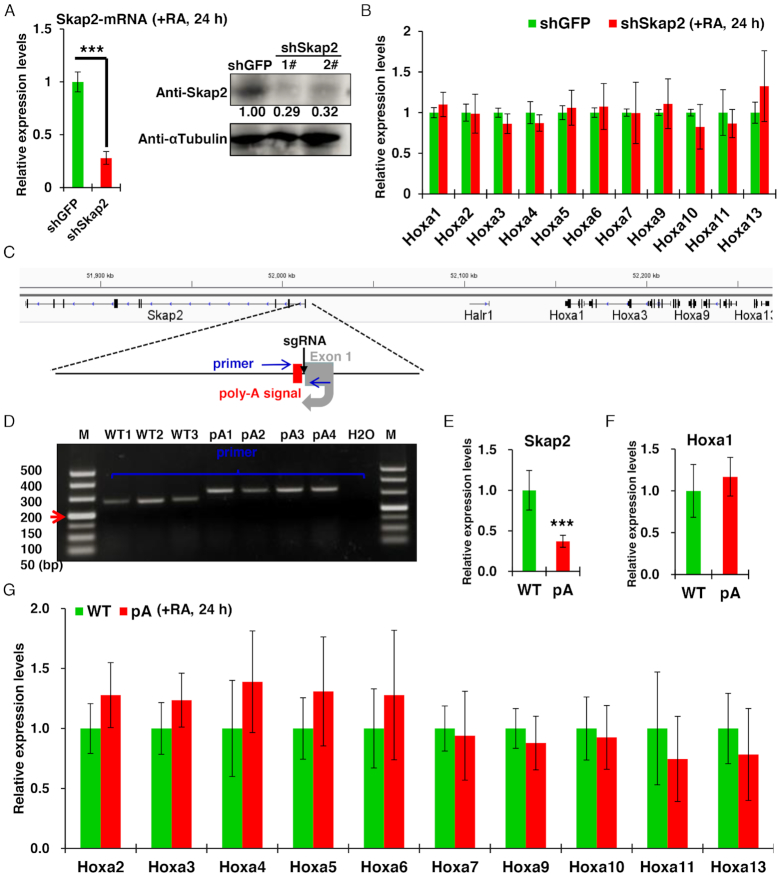
The expression and transcription of Skap2 are not required for Hoxa1 expression. (**A**) Skap2 mRNA and protein levels were significantly inhibited following Skap2 shRNA relative to shGFP control treatment. While Hoxa1 and Hoxa2–a13 expression levels were unchanged (**B**). (**C**) Schematic showing CRISPR/Cas9-mediated insertion of a 49-bp synthetic poly-A signal (red box) downstream of the Skap2 TSS. Primers used for genotyping and Sanger sequencing are shown in blue arrows. (**D**) Genotyping based on gDNA PCR of DNA isolated from indicated WT or poly-A knock-in (pA) cell lines. (**E**–**G**) qRT-PCR of WT and poly-A knock-in (pA) cells showing expression of Skap2 (E), Hoxa1 (F) and Hoxa2–a13 genes (E) in ESCs after RA treatment. Data are represented as mean values ± s.d. Indicated significance is based on Student's *t*-test (*P < 0.05, **P < 0.01, ***P < 0.001). In (A and B), *n* = 3 or 6, including one shRNA for GFP knockdown, two shRNAs for Skap2 knockdown and three technical replicates per cell line. In (E–G), *n* = 9 or 12, including three WT, four Skap2-pA cell lines and three technical replicates per cell line. Image J software was used to quantitative relative protein levels in (A). M: DNA Marker.

Because the Hoxa1 enhancer partially overlaps with the Skap2 TSS, Skap2 expression is lost in EN-KO cells. Previous study has shown that Skap2 is required for β2 integrin-mediated neutrophil recruitment and function (60). Whether Skap2 also regulates Hoxa1 expression and RA-induced ESCs differentiation remains unknown. To distinguish Skap2 function from that of the Hoxa1 enhancer, we first used shRNAs to silence Skap2 expression in ESCs. After 24 h of differentiation of ESCs induced by RA, the mRNA expression and protein levels of Skap2 decreased significantly compared with control cells (Figure 3A). However, expression of Hoxa1–a13 was not changed in Skap2 knockdown and control ESCs (Figure 3B). Thus, we conclude that the expression of Skap2 does not regulate Hoxa1 expression.

Previous studies show that transcriptional activity of adjacent genes may have regulatory effects on neighboring genes (38,61). Although our data indicate that Skap2′ expression does not regulate Hoxa1, we could not exclude the possibility that transcription of Skap2 itself modulated Hoxa1 expression in an unknown manner. Thus, in order to block Skap2 transcription in ESCs, we inserted a 49 bp poly-A termination signal at downstream of the Skap2 TSS by CRISPR/Cas9-mediated knock-in (62) (Figure 3C and D). After 24 h of RA induction, expression levels of Skap2 mRNA in these cells significantly decreased (Figure 3E), indicating that insertion of a poly-A termination signal successfully blocked Skap2 transcription. However, we did not observe significant changes in expression of Hoxa cluster genes in WT versus poly-A-inserted cells (Figure 3F and G), confirming that transcription of Skap2 also has no regulatory effect on Hoxa1 expression.

### Hoxa1 enhancer knockout inhibits RA induction of endoderm master control genes

**Figure 4.**
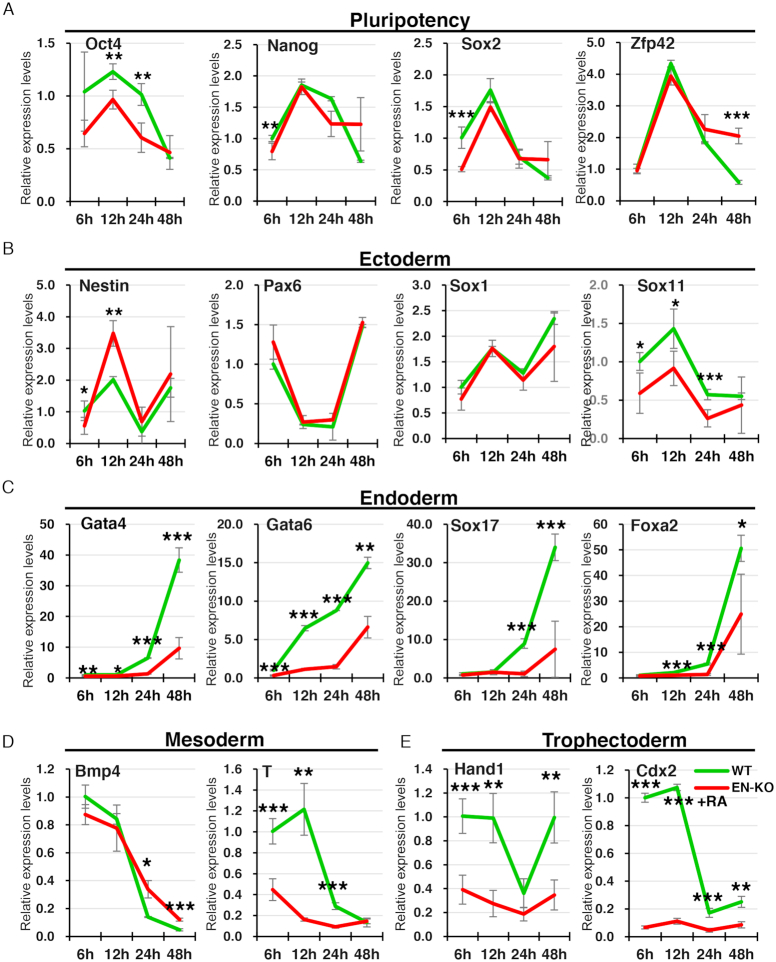
Hoxa1 enhancer knockout inhibits RA induction of endoderm master control genes. (**A**–**E**) Dynamic expression of pluripotency and differentiation master control genes in WT and EN-KO cells over the course of RA-induced ESCs differentiation. mRNAs were measured by qRT-PCR and normalized to Gapdh levels. Data are represented as mean values ± s.d. Indicated significance is based on Student's *t*-test (*P < 0.05, **P < 0.01, ***P < 0.001). In (A–E), *n* = 3 or 6, including one WT, two EN-KO cell lines and three technical replicates per cell line.

Given that Hoxa1 enhancer knockout significantly inhibits Hoxa cluster genes expression, we asked whether RA-induced ESCs differentiation was perturbed by Hoxa1 enhancer loss. To do so, we examined transcript levels of pluripotency and differentiation master control genes in EN-KO versus WT cells after RA induction. Oct4 expression was significantly inhibited in EN-KO relative to WT cells (Figure 4A). Expression of the neuroectodermal gene Nestin increased significantly in EN-KO relative to WT cells, but only at 12 h, while Sox11 expression was relatively lower in EN-KO cells at 6, 12 and 24 h after RA induction (Figure 4B). By contrast, expression levels of Pax6 and Sox1 were comparable in WT and EN-KO cells (Figure 4B). Unexpectedly, high expression of the endoderm master control genes Gata4, Gata6, Sox17 and Foxa2 induced by RA was significantly inhibited in EN-KO relative to WT cells (Figure 4C). Previous studies report that Gata4 and Gata6 are essential for heart development (4,6,63–67), and Hoxa1 mutant mice develop heart disease (18), suggesting a previously uncharacterized association between Hoxa1 and Gata4/6. We also found that mesoderm (T) and trophoblast ectoderm (Hand1, Cdx2) genes expression was blocked in EN-KO relative to WT cells (Figure 4D and E). In addition, we found that Skap2 expression and transcription did not regulate Hoxa1 expression, but whether it affected RA-induced early ESCs differentiation remains unknown. We also detected differentiation-associated master regulatory genes in Skap2 knockdown cells, but no significant changes were found (Supplementary Figure 5A). And the inhibition of Skap2 transcription by poly-A insertion also did not affect the expression of differentiation-associated master regulatory genes (Supplementary Figure 5B). These data suggest that the expression and transcription of Skap2 do not regulate early differentiation of ESCs induced by RA. Overall, our findings indicate that the Hoxa1 enhancer is required for RA-induced ESCs early differentiation.

### RA-induced ESCs early differentiation is blocked in EN-KO cells

**Figure 5.**
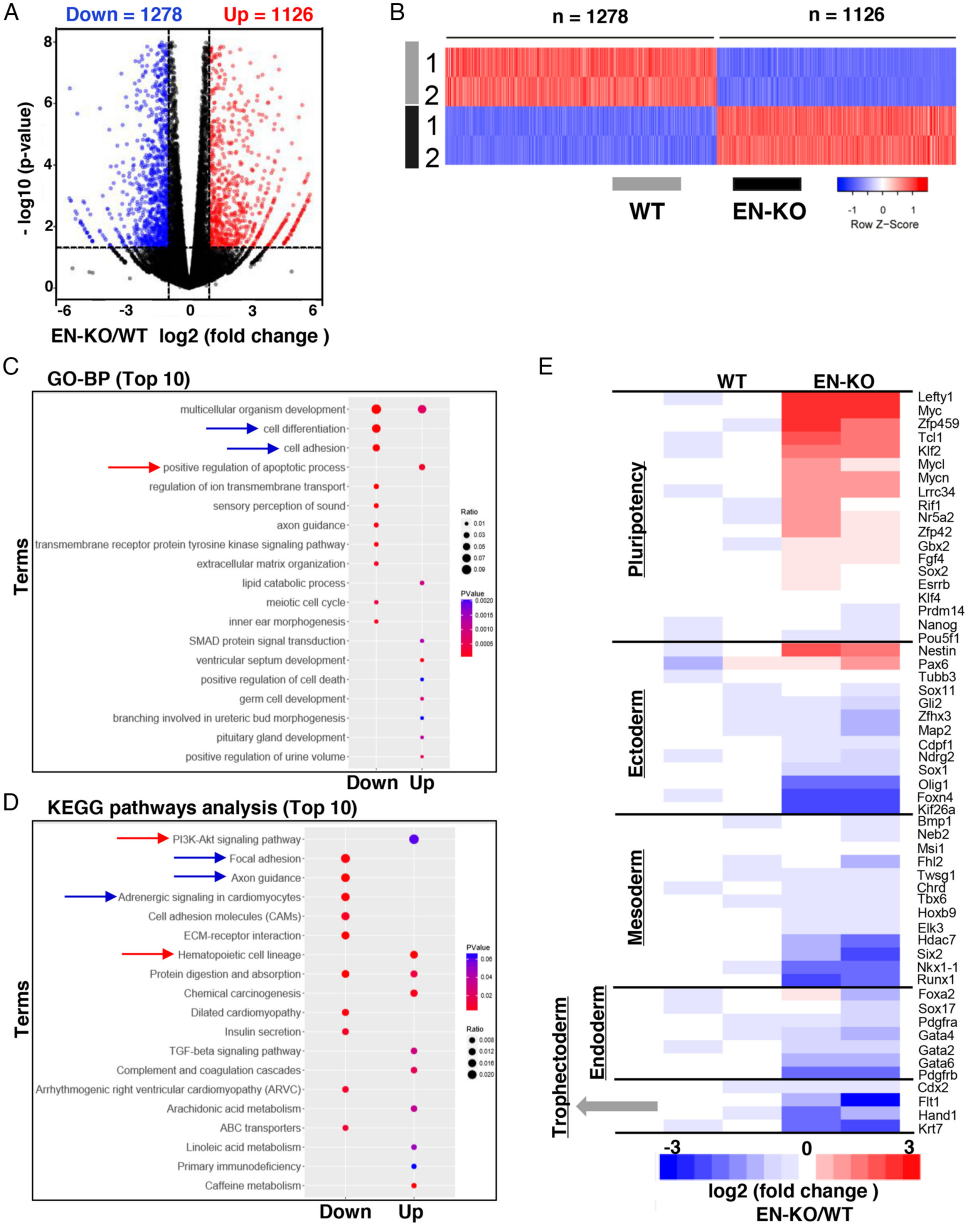
RA-induced ESCs early differentiation is blocked in EN-KO cells. (**A** and **B**) Volcano plot (A) and heatmap (B) depicting gene expression changes in WT and EN-KO ESCs treated with RA (fold-change ⩾ 2, and *P* < 0.05, as determined by DESeq2). (**C** and **D**) GO-BP and KEGG pathway analyses of indicated differentially expressed genes (top 10). (**E**) RNA-seq results shown the Heatmap of pluripotency and differentiation genes.

To assess potential Hoxa1 enhancer function in RA-induced ESCs early differentiation, we performed full transcriptome analysis using RNA-seq. Following 24 h of RA induction in ESCs, we observed downregulation of 1278 genes and upregulation of 1126 genes in EN-KO relative to WT cells (fold change ⩾ 2, *P* < 0.05) (Figure 5A and B). In addition, GO term analysis showed that downregulated genes were significantly enriched in biological processes related to cell differentiation and adhesion (Figure 5C). KEGG signaling pathway analysis also revealed that focal adhesion and myocardial development were enriched in downregulated genes. Upregulated genes were enriched with the PI3K-AKT and hematopoietic cell development signaling pathways (Figure 5D). We then selected pluripotency and differentiation genes to construct a heatmap (Figure 5E) and found that expression of Oct4, Nanog and Sox2 was not significantly changed in EN-KO relative to WT cells; however, non-canonical pluripotency genes (such as Lefty1 and Tcl1) showed significantly increased expression in EN-KO relative to WT cells. Differentiation genes were significantly downregulated in EN-KO relative to WT cells, except for the neurectoderm gene Nestin. These data suggest that Hoxa1 enhancer knockout inhibits differentiation gene expression and promotes non-canonical pluripotency gene expression and that the enhancer is essential for RA-induced ESCs early differentiation.

### Hoxa1 overexpression partially rescues endoderm master genes expression in EN-KO cells

**Figure 6.**
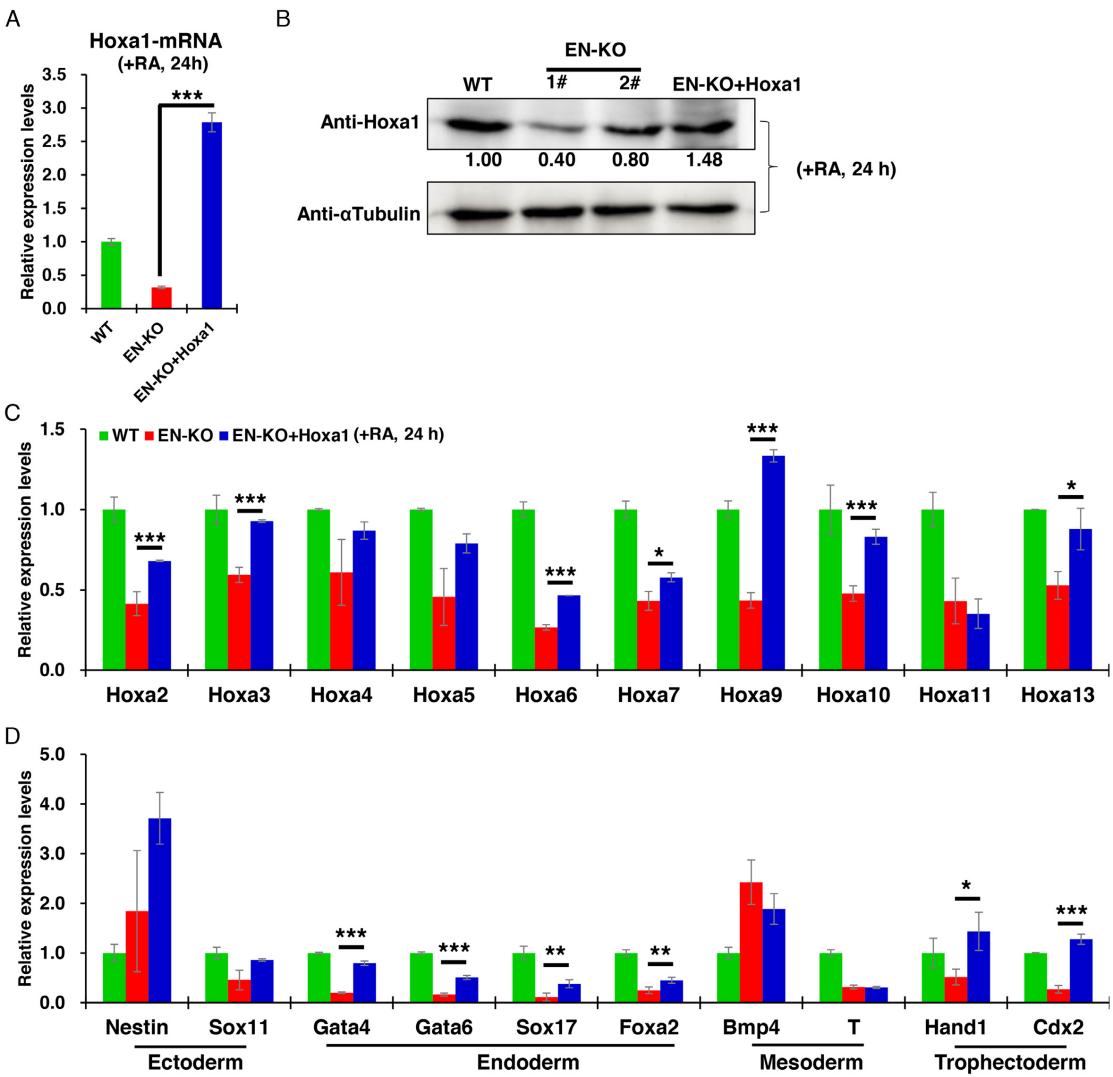
Hoxa1 overexpression partially rescues Hoxa cluster genes and endodermal genes expression in EN-KO cells. (**A**) Hoxa1 mRNA levels in indicated cells were measured over the course of RA treatment by qRT-PCR and normalized to Gapdh levels. (**B**) Hoxa1 protein levels were detected by WB in WT, EN-KO and EN-KO + Hoxa1 cells after RA treatment. Image J software was used to quantitative relative protein levels. (**C**) Hoxa2–a13 mRNA levels in indicated cells were measured over the course of RA treatment by qRT-PCR and normalized to Gapdh levels. (**D**) Expression of indicated differentiation-associated master regulatory genes was measured by qRT-PCR and normalized to Gapdh levels following RA induction. Data are represented as mean values ± s.d. Indicated significance is based on Student's *t*-test (*P < 0.05, **P < 0.01, ***P < 0.001). In (A, C and D), n = 3 or 6, including one WT, two EN-KO and one EN-KO + Hoxa1 cell lines, and three technical replicates per cell line.

To confirm that knockout of the distal enhancer inhibits RA-induced ESCs early differentiation via effects on Hoxa1 expression, we transfected EN-KO cells with a Hoxa1 expression vector and as early as 12 h later detected a significant recovery in Hoxa1 mRNA levels compared with untransfected EN-KO cells (Figure 6A and Supplementary Figure 7A). We also detected the expression of Haxa1 protein in ESCs differentiated by RA for 24 h through western blotting. Compared with EN-KO and wild type cells, the expression of Hoxa1 protein was rescued to a level comparable to wild-type cells (Figure 6B). We had previously found that Hoxa2–a13 and endoderm master control genes transcripts were significantly decreased in EN-KO relative to WT cells. However, when Hoxa1 expression was restored, Hoxa2–a13 expression levels showed a partial recovery over the course of RA-induced ESCs differentiation (Figure 6C and Supplementary Figure 6). Expression of the endoderm master control genes Gata4 and Gata6 were also partially but significantly rescued. However, other differentiation genes did not recover significantly following Hoxa1 overexpression over the course of RA-induced differentiation, with except for trophectoderm genes (Hand1 and Cdx2) (Figure 6D and Supplementary Figure 7). These data indicate that Hoxa1 enhancer loss decreases expression of Hoxa cluster and endoderm master control genes, some of which can be partially rescued by Hoxa1 overexpression, confirming that the enhancer regulates RA-induced ESCs early differentiation by directly controlling Hoxa1 expression.

### Hoxa1 overexpression rescues target genes expression by directly binding to promoters

**Figure 7.**
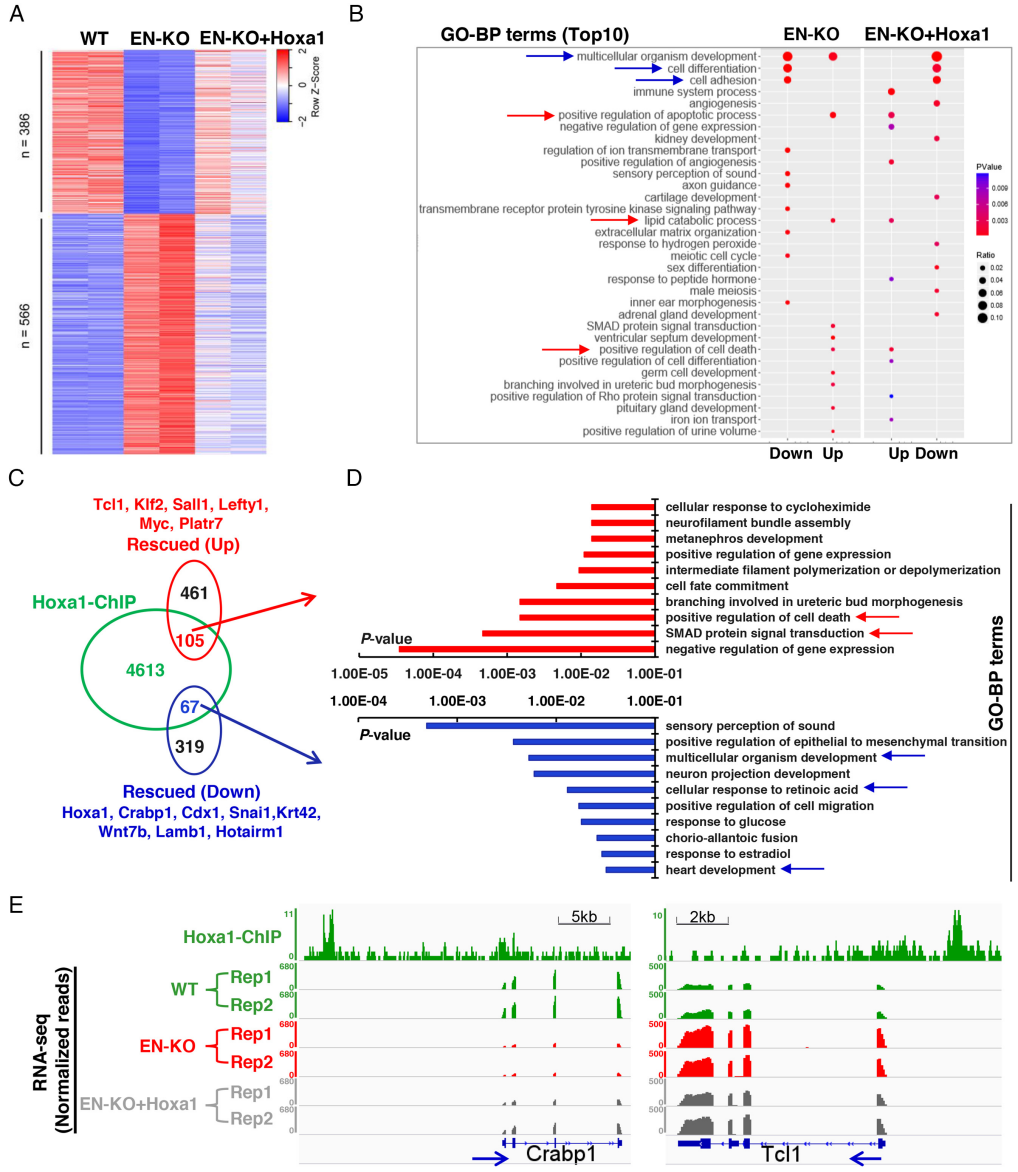
Hoxa1 expression rescues target genes expression through direct binding to promoters. (**A**) Heatmap depicting gene expression changes in WT, EN-KO and EN-KO + Hoxa1 cells following RA treatment (24 h). Shown in EN-KO and EN-KO + Hoxa1 cells are expression levels normalized to mean expression in WT cells. Rescued genes in EN-KO+Hoxa1 cells were determined using *P* < 0.05 compared with EN-KO cells. (**B**) GO analysis showing between EN-KO and EN-KO+Hoxa1 cells following rescue with Hoxa1 expression. (**C**) Overlap of genes whose promoters are directly bound by Hoxa1 based on Hoxa1 ChIP-seq analysis with Hoxa1-rescued genes. (**D**) GO analysis of Hoxa1-rescued genes from (C) (top10). (**E**) IGV screenshots show Hoxa1 binding and RNA-seq at loci of Crabp1 and Tcl1 target genes. Blue arrows indicate the direction of target genes transcription.

Hoxa1 overexpression restored expression of Hoxa cluster genes and endoderm master control genes in EN-KO cells following RA treatment. To better determine the effect of Hoxa1 enhancer on differentiation, we also performed transcriptional analysis in EN-KO cells with overexpressing of Hoxa1 (Supplementary Figure 8A). RNA-seq analysis showed that expression of 952 (40%) of 2406 genes whose expression was altered by enhancer knockout was significantly rescued. Of the 1278 previously downregulated genes, 386 (30%) of 1278 were rescued, while 566 (50%) of the 1126 upregulated genes were rescued (Figure 7A) in EN-KO cells. Further GO analysis revealed that biological processes related to cell differentiation, cell adhesion, angiogenesis, renal development and cartilage development were significantly enriched following Hoxa1 recovery (Figure 7B). These results confirm that the enhancer regulates RA-induced ESCs differentiation primarily by controlling Hoxa1 expression.

To identify genes directly regulated by Hoxa1, we analyzed Hoxa1-ChIP data in WT cells (23) at 24 h after RA induction (TSS ± 3 kb), and then overlapped those results with genes previously rescued by Hoxa1 in EN-KO cells. That analysis showed that 105 (19%) of 566 upregulated and 67 (17%) of 386 downregulated genes were directly regulated by Hoxa1 (Figure 7C). GO-BP terms analysis of these genes showed that downregulated genes were mainly associated with sensory perception of sound, multicellular organism development, neuronal projection development, heart development and the cellular response to retinoic acid. Genes encoding factors associated with SMAD signaling were significantly enriched in upregulated genes (Figure 7D). These results suggest that Hoxa1 regulates various signaling pathways controlling ESCs differentiation by promoting or blocking expression of target genes that include Crabp1, Cdx1, Snai1, Tcl1, Klf2 and Sall1 (Figure 7E; Supplementary Figure 8B-C and 9A-B).

## DISCUSSION

Hoxa1 is rapidly activated and highly expressed in early stages of RA-induced ESCs differentiation. In this study, we identify a distal enhancer that maintains Hoxa1 expression through a long-range chromatin loop, thereby regulating targets that function in RA-induced early differentiation of ESCs.

**Figure 8.**
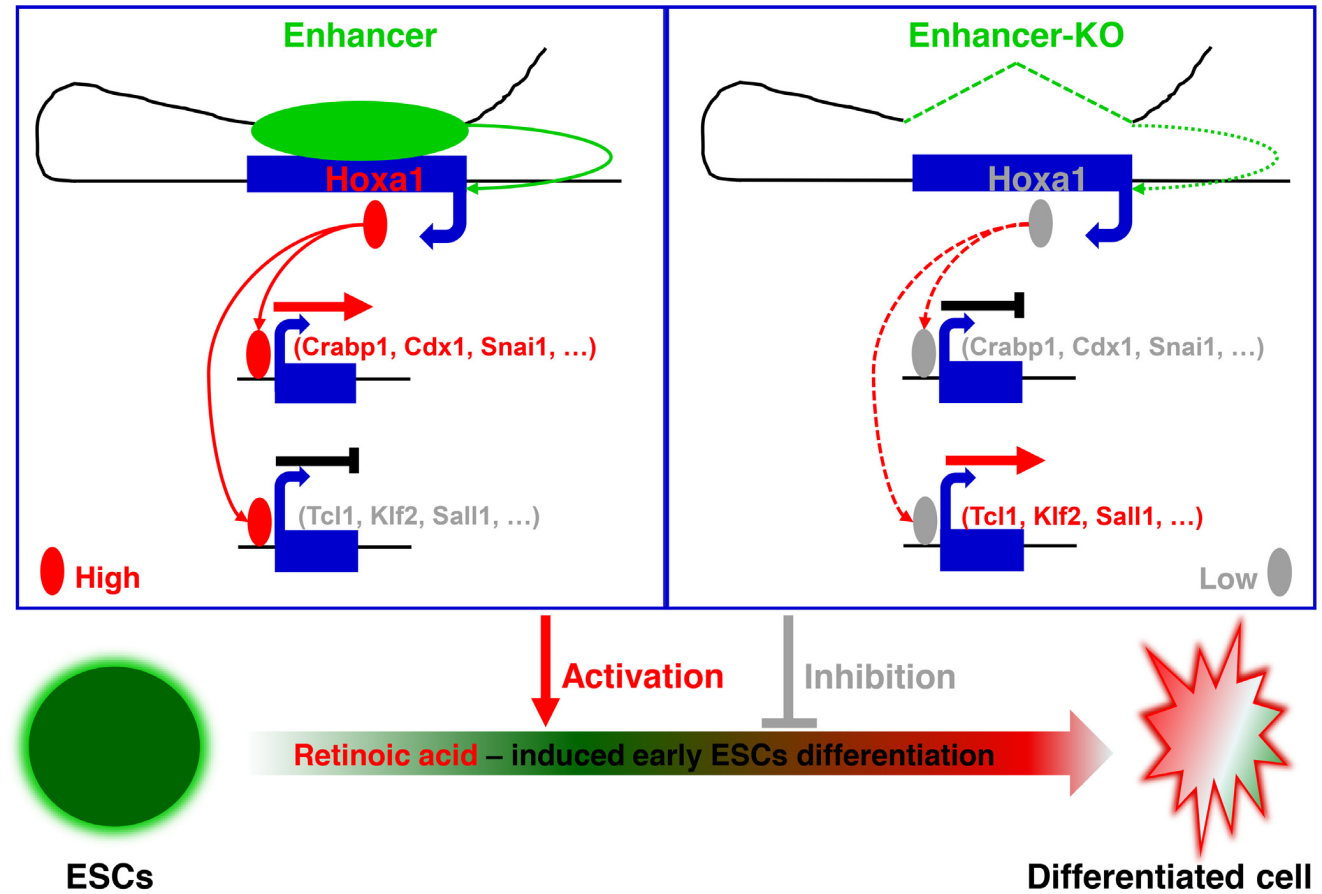
Schematic illustration of a distal enhancer that maintains Hoxa1 expression and orchestrates RA-induced ESCs differentiation. The Hoxa1 enhancer working model is shown: (Left) In WT cells a distal enhancer interacts with the Hoxa1 locus through a chromatin loop to maintain Hoxa1 expression. Hoxa1 protein directly binds to promoters of downstream target genes to either activate (such as Crabp1, Cdx1, Snai1) or inhibit (such as Tcl1, Klf2, Sall1) target genes expression and regulate proper RA-induced early ESCs differentiation. (Right) In enhancer knockout cells, Hoxa1 expression induced by RA is blocked, and appropriate activation or inhibition of downstream target genes are perturbed, resulting in their abnormal expression and impairing RA-induced ESCs early differentiation.

Based on data presented here, we propose the following Hoxa1 enhancer model (Figure 8). In WT cells (left), a distal enhancer interacts with the Hoxa1 locus through a chromatin loop to maintain Hoxa1 expression. Hoxa1 protein then directly binds promoters of downstream genes, where it either promotes (as in the case of Crabp1, Cdx1 or Snai1) or inhibits (as in the case of Tcl1, Klf2 or Sall1) expression of those targets, thereby maintaining proper differentiation of ESCs induced by RA. As evidence, we show that in Hoxa1 enhancer knockout cells (right), RA-induced Hoxa1 expression is inhibited and proper activation or inhibition of downstream targets is perturbed, resulting in abnormal genes expression and disruption of RA-induced ESCs early differentiation.

Based on previous report, six enhancers (HoxA developmental early side 1–6, Ades 1–6) reside at the Skap2 and Hoxa1 loci under induction by Chiron (a Wnt agonist) (68). Knock-out of the Wnt-dependent Ades1 and Ades2 region decreases transcription of Hoxa1 in response to Chiron. Recent studies have also reported two enhancers located downstream of Hoxa1, namely, those we designate the f- and g-site enhancers (Figure 1B). Single or double knock-out of these enhancers significantly represses Hoxa cluster genes expression during RA-induced ESCs early differentiation (27–29). Here, seven enhancers (Figure 1B) were predicted between Skap2 and Hoxa1 loci. Knocking out the e-site enhancer also significantly inhibited Hoxa1 expression and ESCs differentiation in response to RA. These results suggest overall that proper Hoxa1 expression and ESCs differentiation require dynamic changes in enhancer activities via numerous regulatory modes. In addition, activation of enhancers in a chromatin loop plays an important role in regulating target gene expression, and several previously characterized factors mediate these interactions. The best-known are CTCF, cohesin proteins (Smc1a and Smc3) and mediator proteins (Med1 and Med12). However, recent reports identify YY1 as mediating chromatin interaction (69,70). Moreover, long non-coding RNAs (lncRNAs) also function in chromatin interactions by serving as scaffolds for chromatin-binding proteins (71–74). For example, the lncRNA Evx1as RNA regulates Evx1 expression by interacting with mediator proteins and forming a complex that binds to regulatory sites on chromatin, promoting an active chromatin state (75). In our study, we found that the e-site enhancer region and Hoxa1 locus can bind YY1, Med1, Med12, Smc1a and Smc3 (Supplementary Figure 3), suggesting that multiple binding factors may participate in mediating the interactions between the Hoxa1 locus and the Hoxa1 enhancer.

It is well known that lncRNAs and coding genes can regulate neighboring genes expression in numerous modes (38). For instance, transcription of the lncRNA Blustr regulates Sfmbt2 expression. Therefore, because the e-site enhancer overlaps with the Skap2 TSS, we confirmed that their activities were distinct. First, we excluded a role for Skap2 protein in Hoxa1 expression by shRNA-mediated Skap2 knockdown. In addition, in separate analyses we inserted a poly-A termination signal downstream of the Skap2 TSS to block Skap2 transcription. However, expression of Hoxa1 and ESCs differentiation master control genes was unchanged in ESCs cells harboring this construct, indicating that this enhancer controls expression of Hoxa1 and ESCs differentiation independently of Skap2 protein and transcription. A previous study reported that a RARE located at the 3′-end of Hoxa1 is essential for Hoxa1 expression in response to RA. We analyzed Rara-ChIP-seq data (76) and identified three Rara signal peaks in the e-site enhancer region (data not shown). We speculate that these Rara binding sites may serve as RARE for the e-site enhancer maintaining Hoxa1 expression, a possibility that requires further confirmation.

The normal expression of Hoxa1 induced by RA is a key factor for maintaining ESCs’ proper differentiation. In EN-KO cells, Hoxa1 is in a low expression state, leading to arrest of differentiation of ESCs upon RA induction. Subsequently, we restored Hoxa1 expression in EN-KO cells by forcing the expression of exogenous Hoxa1 and found that 40% of the genes were rescued. This result suggests that the e-site enhancer has a certain degree of specificity for Hoxa1 expression. Although multi-sites interactions between e-site enhancer and Hoxa chromatins may also be required for RA-induced Hoxa2–a13 genes expression, whether Hoxa2–a13 can rescue the cellular abnormalities caused by deletion of e-site enhancer may need further exploration.

Proper RA signaling is essential for embryonic development, organogenesis and maintenance of tissue-specific gene expression (77,78). In our RA-induced system, we observed significant increases in expression of endodermal master regulatory genes over the course of ESCs differentiation, consistent with previous reports (76) and suggesting that early RA-induced ESCs differentiation also favors endodermal identity. However, the expression of endoderm master control genes was significantly inhibited in EN-KO cells, and Hoxa1 overexpression in these cells partially rescued Gata4 and Gata6 expression. These results suggest that the Hoxa1 enhancer and Hoxa1 are necessary for RA-induced early endodermal gene expression. We also found that Hoxa1 protein binds to a distal enhancer of Gata4 based on Hoxa1-ChIP-seq data (data not shown) (79). This activity may be important in regulating Gata4 expression. Moreover, a previous study reported that Hoxa1 mutant mice exhibit phenotypes indicative of heart disease (19), confirming a direct association of Hoxa1 with endodermal organ development. However, whether Hoxa1 directly regulates expression of endoderm master control genes (such as Gata4) remains to be demonstrated. Interestingly, we also found that Crabp1 was significantly downregulated in EN-KO cells, and Hoxa1 overexpression partially rescued Crabp1 expression in that context. Hoxa1 protein also significantly binds to the Crabp1 promoter (Figure 7E) and Crabp1 as an RA-binding protein is crucial to activating RA signaling (80,81). Thus, the new cyclic regulation may occur as follows: RA enters cells, Crabp1 transfers RA to RAR/RXR to form a complex, that complex binds to a RARE to promote Hoxa1 expression, newly expressed Hoxa1 protein binds to the Crabp1 promoter and maintains Crabp1 expression, and finally, activation of RA signaling promotes ESCs differentiation. In summary, our results indicate that a novel Hoxa1 enhancer is essential for RA-induced ESCs early differentiation primarily through direct regulation of Hoxa1 expression. This discovery will increase our understanding of gene regulation and the underlying mechanism of ESCs differentiation.

## DATA AVAILABILITY

Capture-C data and RNA-seq data are deposited in the NCBI Gene Expression Omnibus (GEO, https://www.ncbi.nlm.nih.gov/geo/) under the accession number GSE124306.

## Supplementary Material

gkz482_Supplemental_Files
