# Supplementary material for: A distal enhancer maintaining Hoxa1 expression orchestrates retinoic acid-induced early ESCs differentiation
Source: Nucleic Acids Res. 2019 May 31;47(13):6737–52. doi: 10.1093/nar/gkz482 (PMC6649716; doi:10.1093/nar/gkz482)
Supplement: gkz482_Supplemental_Files [file gkz482_supplemental_files.zip › NAR-01254-X-2019.R1--Supplementary Figures-20190516.pdf]

Supplementary Figure 1

A

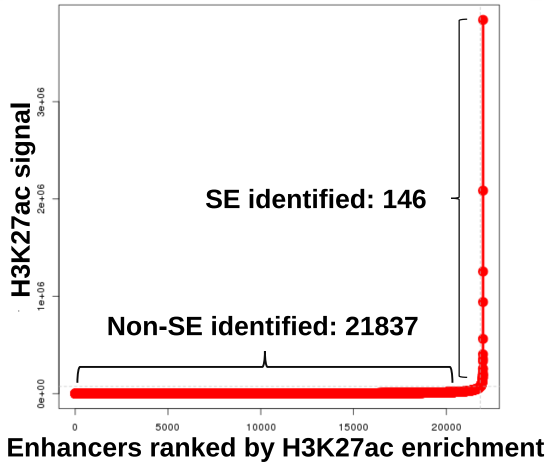

B

Enhancer	Chrom	Start	Stop	H3K27Ac signal	Rank	Size (bp)	SE
**a**	6	51876287	51881926	3301.0706	7097	5640	NO
**b**	6	51925049	51925886	868.8897	13770	838	NO
**c**	6	51947046	51948396	1481.625	11039	1351	NO
**d**	6	51984010	51984947	467.8744	16382	938	NO
**e**	**6**	**52005414**	**52046485**	**39444.5884**	**488**	**41072**	**NO**
**f**	6	52076038	52080034	23963.2128	1005	3997	NO
**g**	6	52103243	52116772	39741.4375	480	13530	NO
**h**	6	52138169	52204406	83716.9443	115	66238	YES
**i**	6	52221403	52234330	3289.9215	7107	12928	NO

Supplementary Figure 1. Predicted enhancers based on H3K27ac signals in RA-treated ESCs. (**A**) Shown is ranking of enhancers (21983) based on H3K27ac occupancy, including 146 super-enhancers (SE). (**B**) Characteristics of nine predicted enhancers at the Skap2 and Hoxa loci. The e-site enhancer is shown in red. (As a supplement to Figure 1)

Supplementary Figure 2

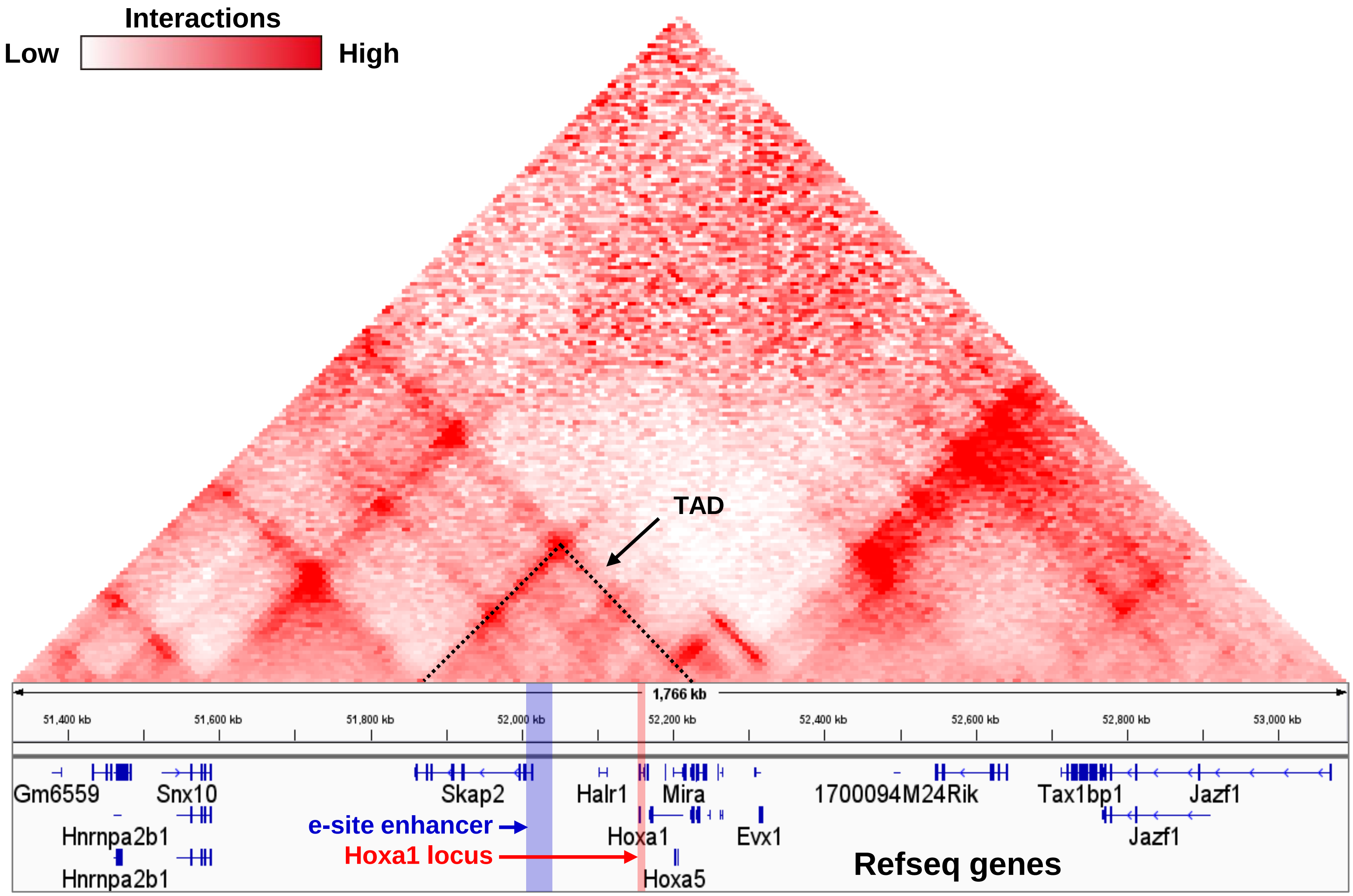

Supplementary Figure 2. Hi-C interaction map of the ~ 1.7 Mb region surrounding the Hoxa1 in undifferentiation ESCs. Data were extracted from Bonev B et al. 2017. Black dotted line indicates the predicted TAD (mm10 chr6:51880000-52240000). Blue shadow region indicates e-site enhancer locus. Red shadow region indicates Hoxa1 locus. (As a supplement to Figure 1)

Supplementary Figure 3

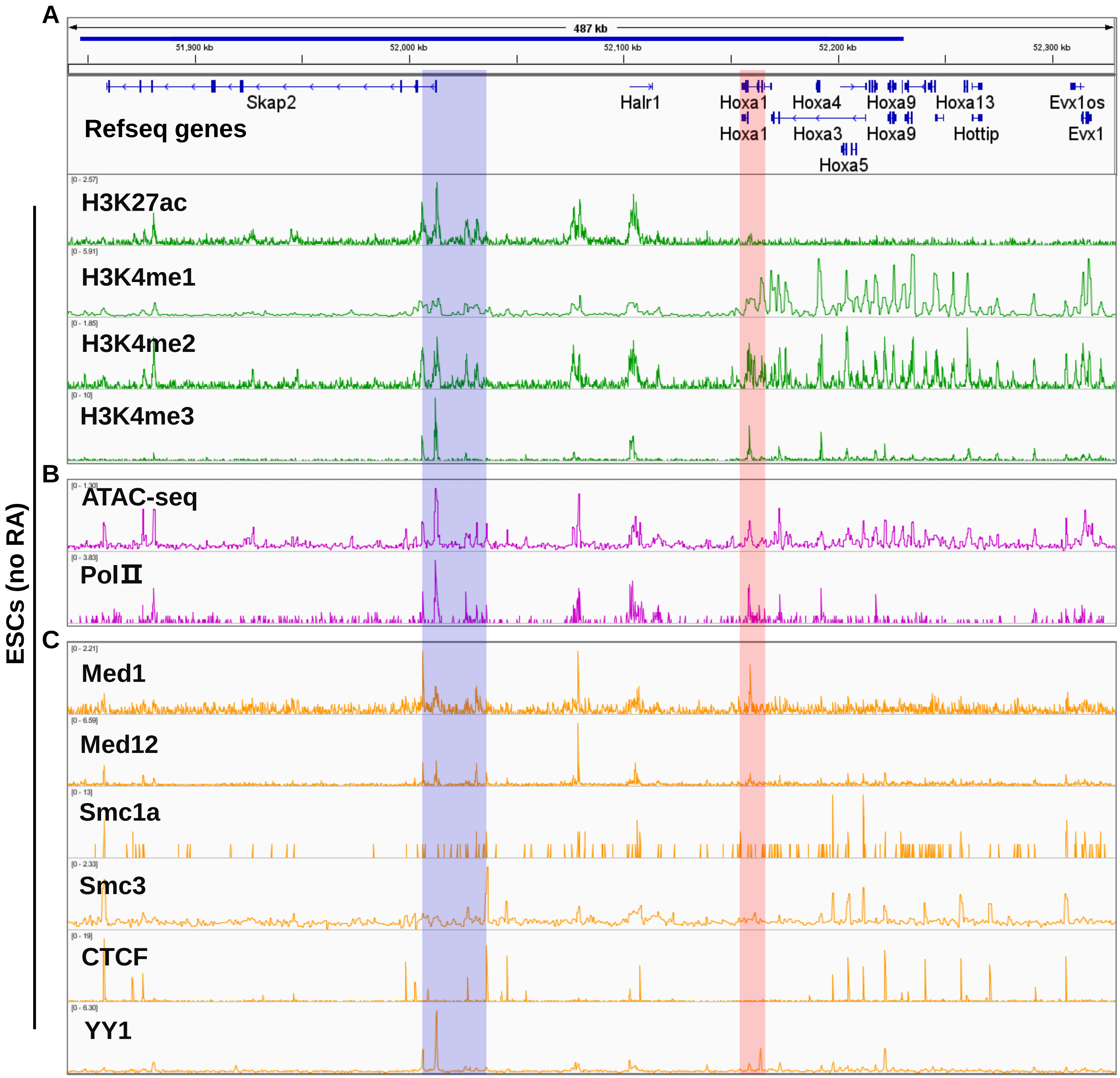

Supplementary Figure 3. IGV view of selected ChIP-seq tracks at Skap2 and Hoxa cluster loci in undifferentiation ESCs. (**A-C**) Shown are H3K27ac, H3K4me1, H3K4me2, H3K4me3, ATAC-seq, PolⅡ, Med1, Med12, Smc1a, Smc3, CTCF and YY1 tracks corresponding to binding sites of indicated factors in undifferentiation ESCs. Blue shadowing indicates e-site enhancer region, red shadowing shows the Hoxa1 locus. Bold blue line indicates predicted TAD (mm10 chr6:51880000-52240000). (As a supplement to Figure 1)

Supplementary Figure 4

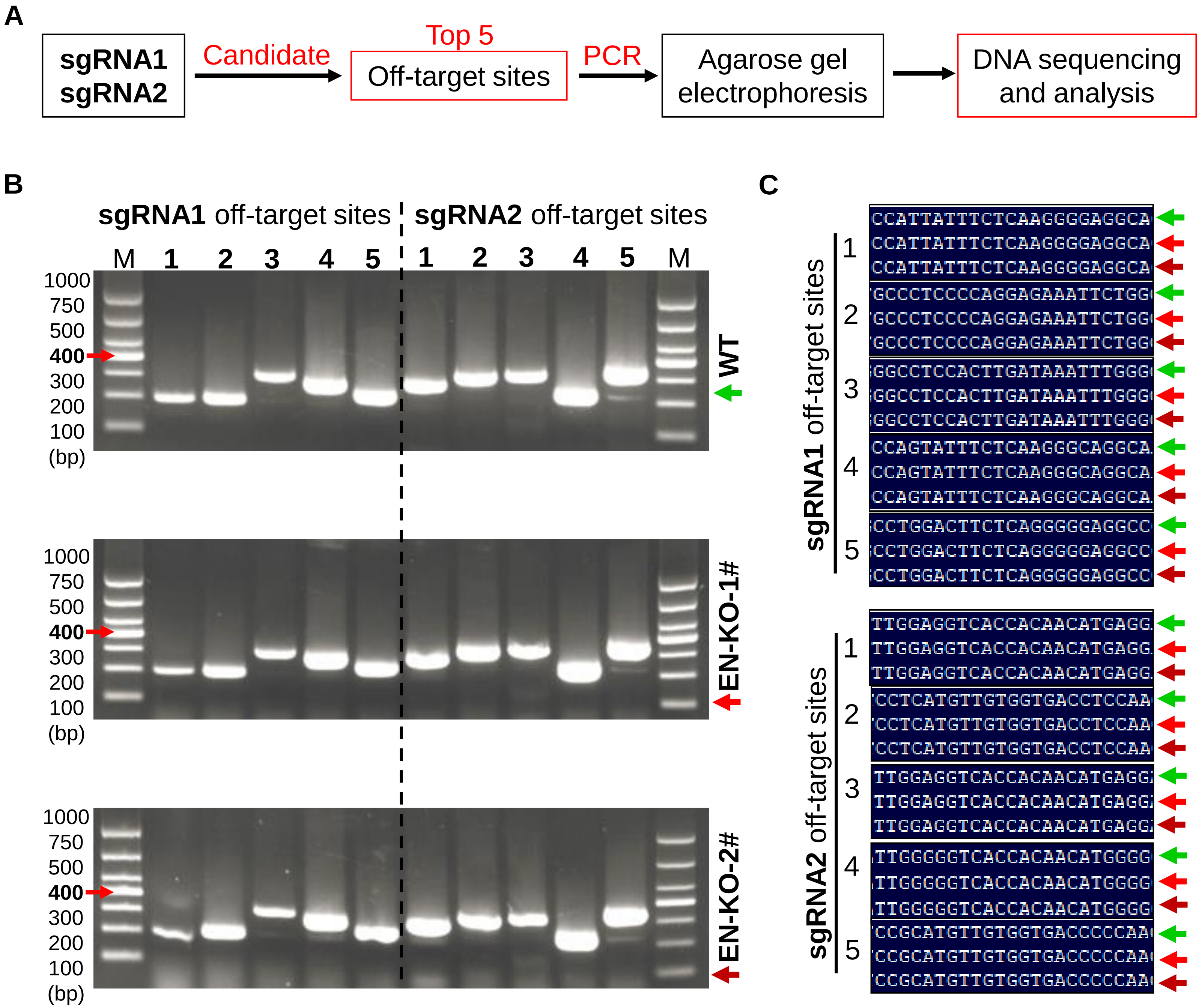

Supplementary Figure 4. Off-target analysis of two sgRNAs used for e-site enhancer knockout. (**A**) Flow chart of off-target analysis (genomic location of sgRNAs off-target sites are shown in Supplementary Table 8). (**B**) Genomic DNA (gDNA) PCR in three cell lines (WT, EN-KO-1#, EN-KO-2#) (primers used for off-target detection are shown in Supplementary Table 9). (**C**) Sequences alignment results showed that no off-target genetic alterations were detected. M: DNA Marker. (As a supplement to Figure 2)

Supplementary Figure 5

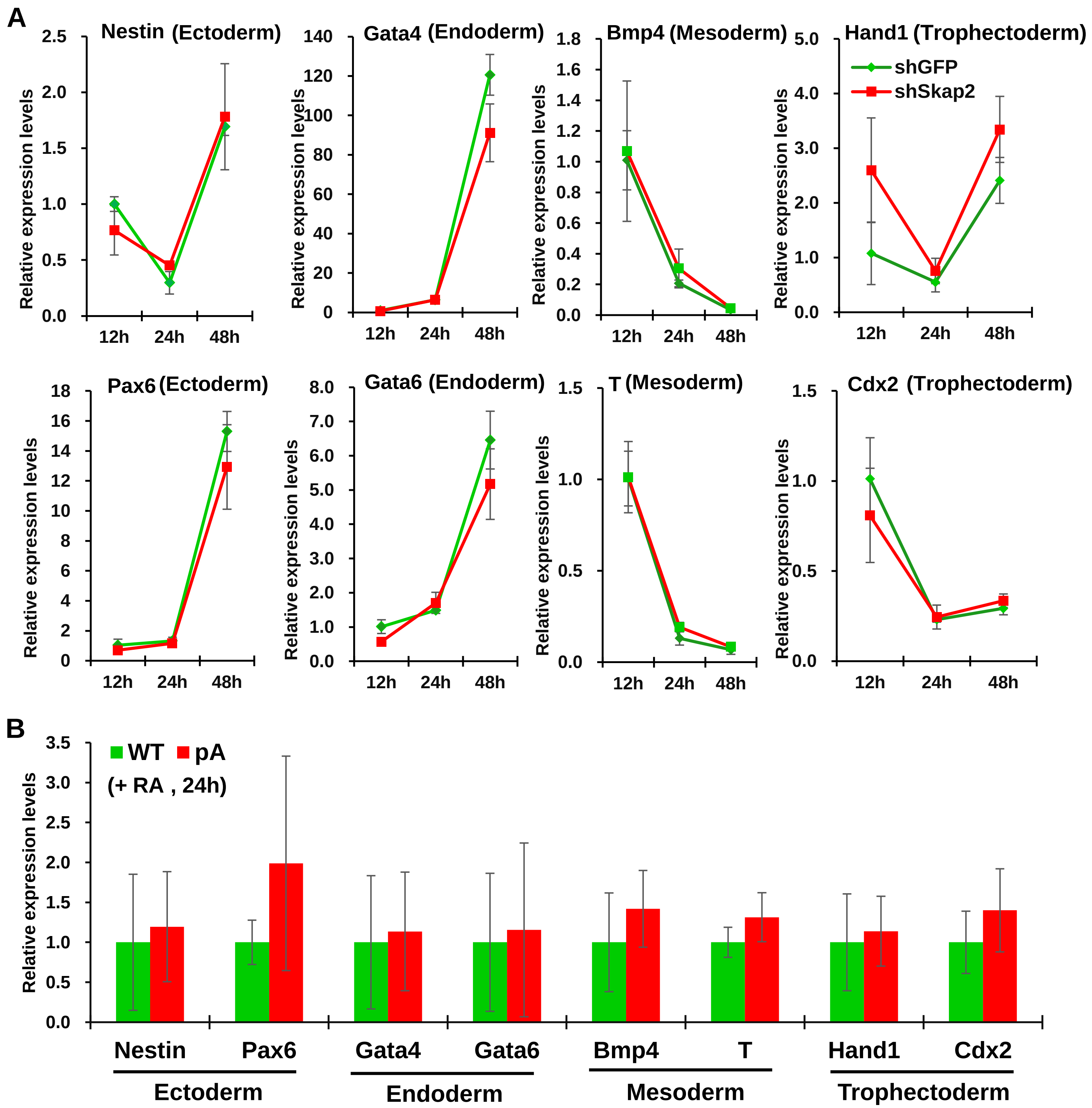

Supplementary Figure 5. Expression and transcription of Skap2 are not required for RA-dependent early ESCs differentiation. (**A**) Differentiation-associated master control genes were measured by qRT–PCR following RA induction in Skap2 knockdown and control cells. (**B**) Following RA induction (24 h), expression levels of differentiation-associated master control genes were also measured by qRT–PCR in WT and Skap2-pA cells. In (**A**), n = 3 or 6, including 1 shRNA for GFP knockdown, 2 shRNAs for Skap2 knockdown, and 3 technical replicates per cell line. In (**B**), n = 9 or 12, including 3 WT and 4 Skap2-pA cell lines, and 3 technical replicates per cell line. Data are shown as mean values± s.d. (As a supplement to Figure 4)

Supplementary Figure 6

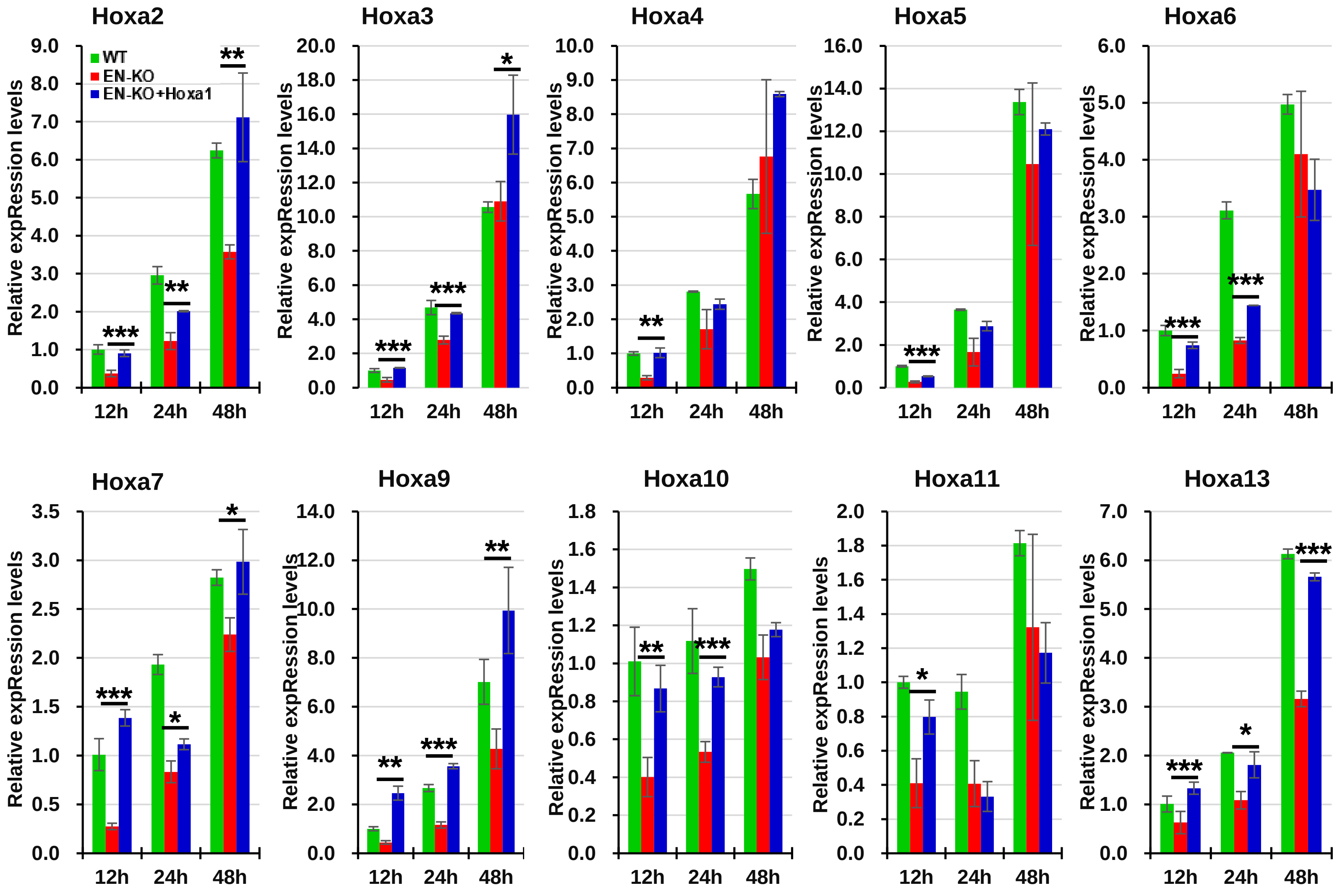

Supplementary Figure 6. Hoxa1 overexpression partially rescues Hoxa genes expression in EN-KO cells. qRT-PCR analysis of Hoxa cluster genes expression at indicated time points after RA treatment in WT, EN-KO or EN-KO+Hoxa1 cells. Data are represented as mean values ± s.d, and indicated significance was determined based on Student's *t*-test (* $P < 0.05$, ** $P < 0.01$, *** $P < 0.001$). (n = 3 or 6, including 1 WT, 2 EN-KO and 1 EN-KO+Hoxa1 cell lines, and 3 technical replicates per cell line.) (As a supplement to Figure 6)

Supplementary Figure 7

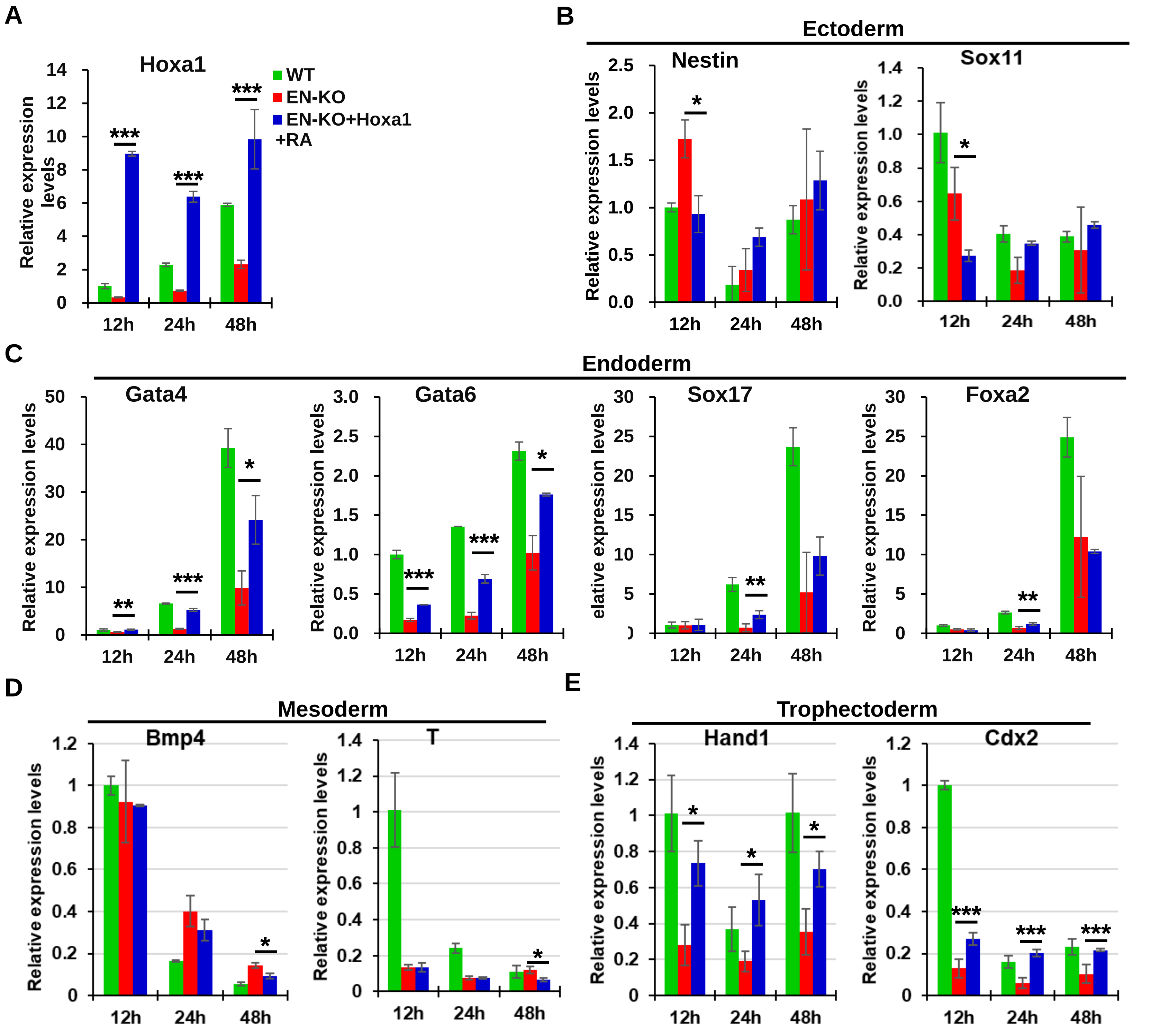

Supplementary Figure 7. Hoxa1 overexpression partially rescues endodermal genes expression in EN-KO cells. (**A**) Hoxa1 mRNA levels in indicated cells were measured over the course of RA treatment by qRT–PCR and normalized to Gapdh levels. Results are shown as mean values ± sd. (**B-E**) Expression of indicated differentiation-associated master regulatory genes was measured by qRT–PCR and normalized to Gapdh levels following RA induction. Data are represented as mean values ± s.d. Indicated significance was based on Student's *t*-test (* $P < 0.05$, ** $P < 0.01$, *** $P < 0.001$). In (**A-E**), n = 3 or 6, including 1 WT, 2 EN-KO and 1 EN-KO + Hoxa1 cell lines, and 3 technical replicates per cell line. (As a supplement to Figure 6)

Supplementary Figure 8

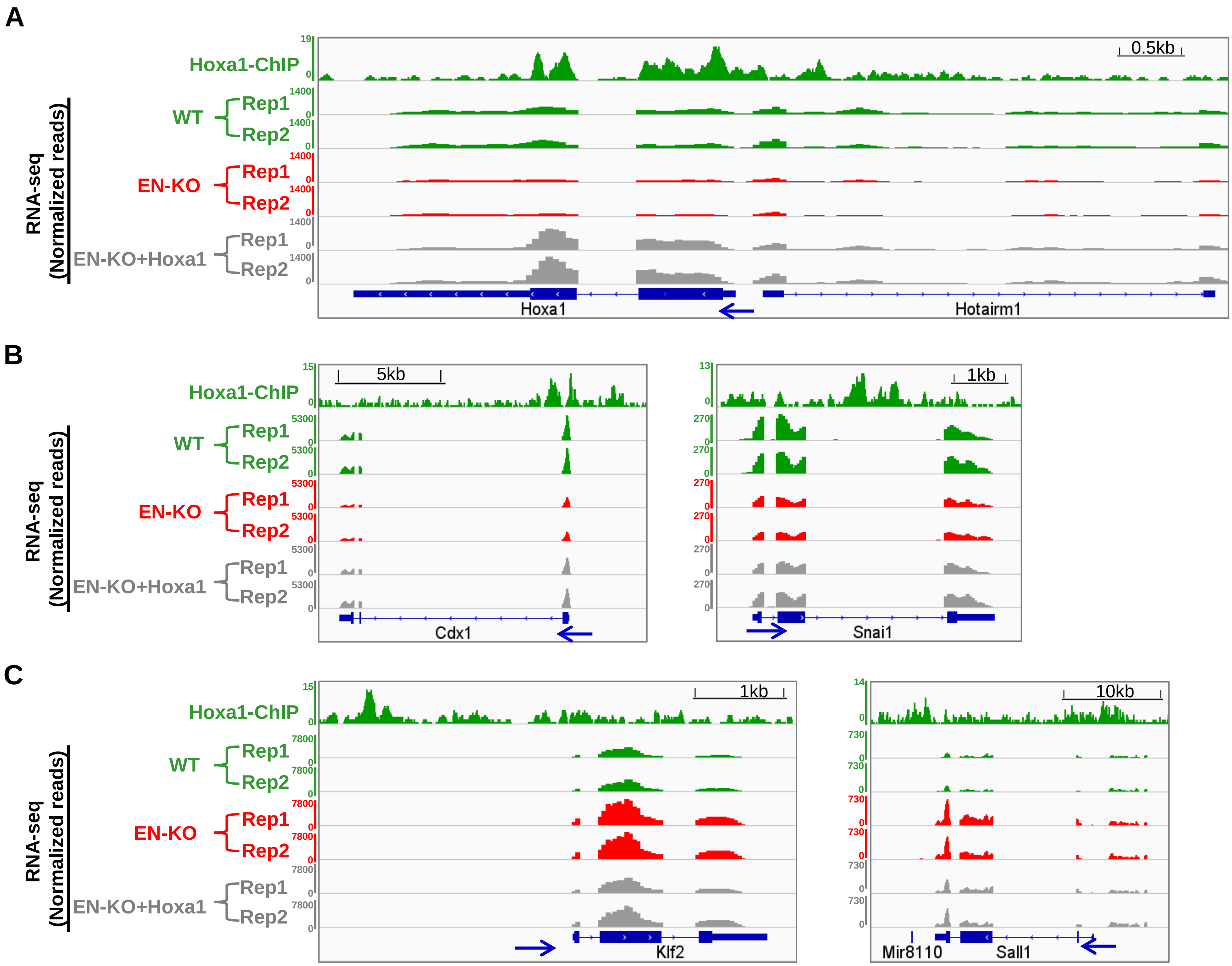

Supplementary Figure 8. IGV screenshots showing Hoxa1 binding and RNA-seq data from various target genes loci. Shown are Hoxa1 (**A**), Cdx1 and Snai1 (**B**), and Klf2 and Sall1 (**C**) loci. Blue arrows indicate direction of target genes transcription. (As a supplement to Figure 7)

Supplementary Figure 9

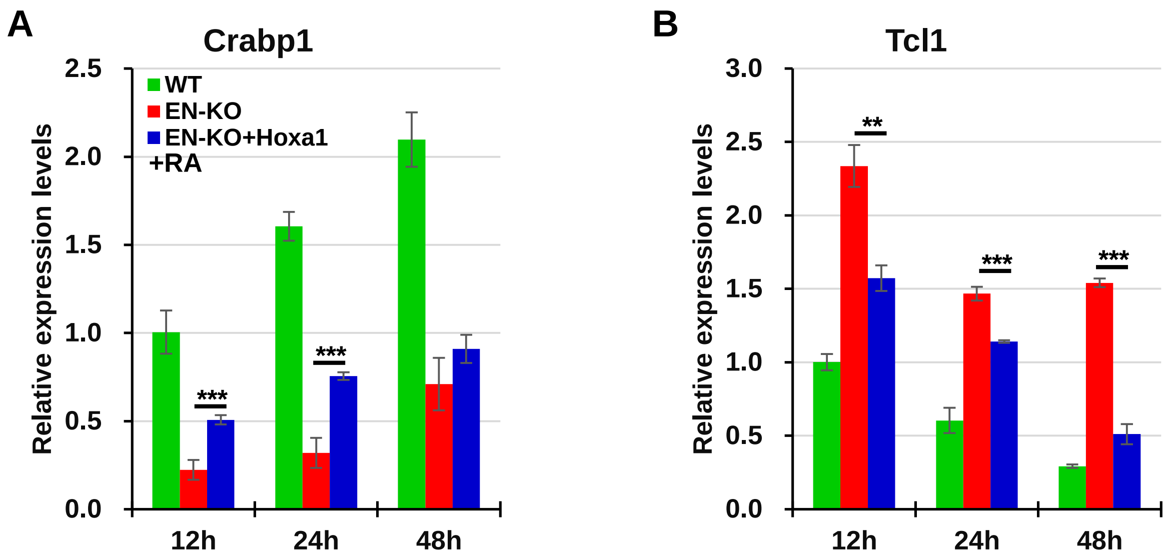

Supplementary Figure 9. Partial rescue of Crabp1 and Tcl1 expression in RA-treated EN-KO cells following Hoxa1 overexpression. (**A** and **B**) qRT-PCR analysis of Crabp1 (**A**) and Tcl1 (**B**) expression at indicated time points under RA treatment in WT, EN-KO and EN-KO+Hoxa1 cells. Data are represented as mean values ± s.d. Indicated significance was determined based on Student's *t*-test (* $P < 0.05$, ** $P < 0.01$, *** $P < 0.001$). In (**A** and **B**), n = 3 or 6, including 1 WT, 2 EN-KO and 1 EN-KO+Hoxa1 cell lines, and 3 technical replicates per cell line. (As a supplement to Figure 7)
