# Supplementary material for: A distal enhancer maintaining Hoxa1 expression orchestrates retinoic acid-induced early ESCs differentiation
Source: Nucleic Acids Res. 2019 May 31;47(13):6737–52. doi: 10.1093/nar/gkz482 (PMC6649716; doi:10.1093/nar/gkz482)
Supplement: gkz482_Supplemental_Files [file gkz482_supplemental_files.zip › NAR-01254-X-2019.R1--Supplementary Tables-2019016.pdf]

Supplementary tables

Supplementary Table 1. Sequences of qRT-PCR primers (5’-3’)

Gapdh	forward	CTACCCCCAATGTGTCCGTC
	reverse	TGAAGTCGCAGGAGACAACC
Hoxa1	forward	CCACCAGGGTTATGCTGGG
	reverse	CGTGGAGAGGGGATAAGGAGTTA
Hoxa2	forward	TACGAATTTGAGCGAGAGATTGG
	reverse	GTCGAGGTCTTGATTGATGAACT
Hoxa3	forward	CCCACAGAAACGCTACACAG
	reverse	GAGTGGCCCAGAGTTGCTC
Hoxa4	forward	ACCCCTGGATGAAGAAGATCCA
	reverse	CGGGTCAGGTAGCGGTTAAAG
Hoxa5	forward	TCAGCCCCAGATCTACCCCT
	reverse	TTTCGATCCTTCTTCGGCGG
Hoxa6	forward	TCCCGAGCAGCAGTACAAAC
	reverse	GTGTCTGGTAGCGCGTGTAG
Hoxa7	forward	CTACGACCAAAACATCCCCG
	reverse	ATTCCTTCTCCAGTTCCAGCG
Hoxa9	forward	GCGCCTTCTCCGAAAACAAT
	reverse	CCAGCGTCTGGTGTTTTGTG
Hoxa10	forward	TTCGTTAGGCCTTTCCGCAT
	reverse	GCTGCATTTTCGCCTTTGGA
Hoxa11	forward	TCTTCGCGCCCAATGACATAC
	reverse	GGCTCAATGGCGTACTCTCT
Hoxa13	forward	GGAACGGCCAAATGTACTGC
	reverse	CTGAAGGATGGGAGACGACG
Skap2	forward	CAGACACGATTTCCTTAGCCTC
	reverse	GGTAGCCAGCCTTTATGACAAA
Oct4	forward	ATCAGCTTGGGCTAGAGAAGGATG
	reverse	AAAGGTGTCCCTGTAGCCTCATAC
Nanog	forward	TACAAGGGTCTGCTACTGAGATGC
	reverse	TTGGGACTGGTAGAAGAATCAGGG
Sox2	forward	GCGGAGTGGAAACTTTTGTCC
	reverse	CGGGAAGCGTGTACTTATCCTT
Zfp42	forward	CCCTCGACAGACTGACCCTAA
	reverse	TCGGGGCTAATCTCACTTTCAT
Nestin	forward	CTGGATCTGGAAGTCAACAGAGGT
	reverse	ATC CTC AGT TTC CAC TCC TGT AGC
Pax6	forward	GCGCAGACGGCATGTATGATA
	reverse	GGGTTGCCCTGGTACTGAAG
Sox1	forward	GAAATCCCACCAAGCGGAGT
	reverse	GGGCCATGGCAGTTAAAACG

Sox11	forward	ACTTTGCAACTTGCCGGAGA
	reverse	CGTTCATGGGCCGTTTGATG
Gata4	forward	GCTATGCATCTCCTGTCACTCAGA
	reverse	CCAAGTCCGAGCAGGAATTTGAAG
Gata6	forward	CTTCTCCTTCTACACAAGCGACCA
	reverse	ATACTTGAGGTCACTGTTCTCGGG
Sox17	forward	GATGCGGGATACGCCAGTG
	reverse	CCACCACCTCGCCTTTCAC
Foxa2	forward	CAGGTCATGCACTACCCAGG
	reverse	GGGCCTGAAAGCCATCTTCT
Bmp4	forward	ACAGCGGTCCAGGAAGAAGAAT
	reverse	TGCACAATGGCATGGTTGGT
T	forward	CATCGGAACAGCTCTCCACCTAT
	reverse	TACCATTGCTCACAGACCAGAGAC
Hand1	forward	AAGGATGCACAAGCAGGTGAC
	reverse	TTTAATCCTCTTCTCGCCGGG
Cdx2	forward	CAGTCCCTAGGAAGCCAAGTGAAA
	reverse	AAGTGAAACTCCTTCTCCAGCTCC
Tcl1	forward	CCTTGGGGGAAGCTATGTCC
	reverse	AGCTGCAGGACCTTGATATGG
Crabp1	forward	CAGCAGCGAGAATTTCGACGA
	reverse	CGCACAGTAGTGGATGTCTTGA

Supplementary Table 2. shRNA sequences (5’-3’)

shGFP	TACAACAGCCACAACGTCTAT	
shSkap2	1#	CACTGTGATTGTACGTGAATC
	2#	GATGGGTTACATTAGATACAA

Supplementary Table 3. sgRNA sequences (5’-3’)

e-site enhancer knockout	sgRNA1	GGCCTCCCCTTGAGAAATTC
	sgRNA2	TTGGAGGTCACCACAACGTG
poly-A signal knock-in	sgRNA	CCTCACCCTATGCGGACGGT

Supplementary Table 4. Knockout and knock-in identification primers

e-site enhancer knockout	primer1	forward	TGGCCTCCTAACTCCCAACA
		reverse	ATGTGCCCGGCTGAATTGAT
	primer2	forward	GAAAATGGGACCCCATCCCT
		reverse	CCCTGAGAAGCTGCCCTAAC
poly-A signal knock-in	primer	forward	GTGCTTACTCGGACCGCTAT
		reverse	CATACACGCCCACTCCCTTC

Supplementary Table 5. Synthetic oligos for Skap2 poly-A knock-in (5'-3')

poly-A knock-in	TTAGCCCTCACCCTATGCGGACGGTAATAAAAGATCTTTATTTTCATTAGATCTGTGTGTTGGTTTTTTGTGTGGGGGGGCGGGCCGTATAGGGGGACT

Note: The yellow shadow marked DNA sequence is the 49 bp poly-A signal. The green shadow marked DNA sequence is NGG. The red shadow marked DNA sequence is sgRNA sequences.

Supplementary Table 6. Primer pairs used for *Hoxa1* vector construction

Hoxa1	forward	CTAGCTAGCATGGACAATGCAAGAATGAACT
	reverse	ATTTGCGGCCGCGTGGGAGGTAGTCAGAGTGTCT

Supplementary Table 7. Synthetic oligos for Capture-C (5'-3') (5'-biotin).

Hoxa1 Capture-C	probe1	GATCAGGCTCGTGATTAATGGAGCCTTGTAGATATTAACAGGACATCACCATCACCACCATCACCACCATCACCACCTCCCACTCCCCGC
	probe2	GAAATCCCAAGGTTTCCAGAAGTCTGGTAAGTAGCCGTCTGGGGGTGGTGATGGTGGTGGTGGTGGTGGTGGTGGTGGGGCGAGCTGATC
e-site enhancer Capture-C	probe1	AGCACATTTCAGCATACACGCCCACTCCCTTCTAGTCCCGACATCCCAGTCCCCCTATACGGCCCGCCCCCCACCGTCCGCATAGGGTGA
	probe2	TTCTGTCTTTTTTATTTGTACTCCATTCATTCCACAAAGGAATTGAGGTGATTTGCTCTGTATCAAATAGACAGCTCTCTCGAGTAGATC

Supplementary Table 8. Genomic location of sgRNAs off-target sites.

	Top5	Genomic location of off-target sites	Sequences
sgRNA1	1	chr1:174148742	tGCCTCCCCTTGAGAAATaaTGG
	2	chr10:121444346	GcCCTCCCCagGAGAAATTCTGG
	3	chr13:83698128	CCCaAATTTaTCAAGtGGAGGCC
	4	chr14:87529483	CCAGtATTTCTCAAGGGcAGGCa
	5	chr19:5645710	CCTGgAcTTCTCAgGGGGAGGCC
sgRNA2	1	chr1:4147423	TTGGAGGTCACCACAACaTGAGG
	2	chr1:12062952	CCTCAtGTTGTGGTGACCTCCAA
	3	chr1:35529213	TTGGAGGTCACCACAACaTGAGG
	4	chr1:37919817	TTGGgGGTCACCACAACaTGGGG
	5	chr1:39682356	CCGCAtGTTGTGGTGACCcCCAA

Supplementary Table 9. Primers used for off-target detection.

	Top5	Sequences	
sgRNA1	1	forward	TCCCATGGAGAAAAATCAATTCC
		reverse	TCTGCTGGATGCTTCCCATT
	2	forward	TGTGCACGATGCTTGGTAGA
		reverse	GCCATGCCAGAATTTCTCCT
	3	forward	TGTATACCAGACTGACCGTGAA
		reverse	TTTCTGCCGGAAAAGGAGACC
	4	forward	CTGGACGCTAGGTTAACTCATTT
		reverse	GCAATGGGCAGCATGAGAAA
	5	forward	CGTATTGCTGTGCCTACCCG
		reverse	CTGTGACAAGAGCCCTGGAT
sgRNA2	1	forward	AGTATGCCAGGTATTGGGGC
		reverse	GACATGCTCCTAATGAACTCAGC
	2	forward	CTGTGGTTGGCACCAACTTTC
		reverse	TAAGGGAATCGAGGCCAGAGA
	3	forward	TCTGGGTCCTGACATGAGGT
		reverse	TAATGGTGGGTCGGTTGTGG
	4	forward	AGCAGTGGTTATCTACCTGTGTG
		reverse	GGTTTAAGGCTGAGACCGACA
	5	forward	TCCAGGGGACAATGACAAGG
		reverse	GTAGCCGACATGTTAGGCCC
